# Somatostatin receptor 4 (SSTR4) is a tumor suppressor in cutaneous and head & neck squamous cell carcinomas

**DOI:** 10.1002/1878-0261.70316

**Published:** 2026-08-07

**Authors:** Ali Taqvi, Tuan Khang Huynh, Adway Kadam, Zahra Kardan, Yue Yan, Amina Jamal Laham, Sampath Kumar Loganathan

**Affiliations:** ^1^ Division of Clinical and Translational Research, Faculty of Medicine and Health Sciences McGill University Montreal Canada; ^2^ Cancer Research Program Research Institute of the McGill University Health Centre Montreal Canada; ^3^ Department of Surgical and Interventional Sciences, Faculty of Medicine and Health Sciences McGill University Montreal Canada; ^4^ Department of Otolaryngology, Head and Neck Surgery, Faculty of Medicine and Health Sciences McGill University Montreal Canada; ^5^ Department of Biochemistry, and Rosalind and Morris Goodman Cancer Research Institute, Faculty of Medicine and Health Sciences McGill University Montreal Canada

**Keywords:** CRISPR/Cas9, cutaneous squamous cell carcinoma, head and neck squamous cell carcinoma, MAPK/ERK pathway, somatostatin receptor 4, SSTR4

## Abstract

The mutational landscape of head & neck and cutaneous squamous cell carcinomas (HNSCC, cSCC) has major gene alterations involving *PIK3CA*, *NOTCH1*, and *TP53* among others that contribute to tumorigenesis. In this study, we focused on somatostatin receptor 4 (*SSTR4*), which was identified in a recent *in vivo* HNSCC CRISPR screen. Mutations in the SSTR protein family are more commonly implicated in neuroendocrine tumors, and to date, no studies have shown a link between SSTR4 and cSCC/HNSCC. To understand the role of Sstr4 in skin keratinocytes, we employed transcriptomic profiling and proteomic methods and identified enriched pathways and protein–protein interactions (PPIs) networks. Sstr4 activation by J‐2156 agonist regulated MAPK‐ERK signaling pathway, which simultaneously limited G1 to S phase cell cycle progression. To validate Sstr4 as a driver of cSCC/HNSCC tumorigenesis, we performed a localized and clonal Sstr4 knockout in basal keratinocytes of the Pik3ca^H1047R^ oncogenic mouse model using ultrasound‐guided *in‐utero* lentivirus injection technology. Tumor formation in mice following Sstr4 knockout occurred rapidly around 12 weeks. Our study is the first to uncover Sstr4 role in cSCC/HNSCC through *in‐vivo* models.

## Graphical abstract

This study identifies somatostatin receptor 4 (Sstr4) as a critical tumor suppressor against skin and head/neck cancers (HNSCC, cSCC, and BCC). The loss of Sstr4 removes a check on cell growth, causing hyperactivation of the MAPK‐ERK signaling pathway (↑). We demonstrate that this signaling dysregulation directly triggers rapid tumorigenesis in our heterozygous Pik3ca^H1047R^ oncogenic mutant mouse model.

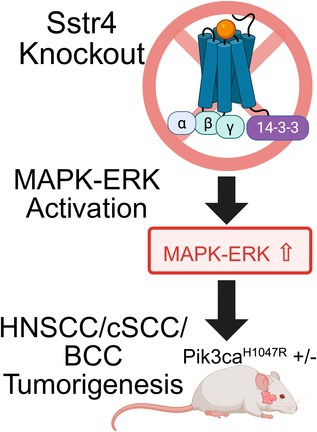

**Abbreviations**
AHR: aryl hydrocarbon receptor
BCC: basal cell carcinoma
cAMP: cyclic adenosine monophosphate
CDKN2A: cyclin‐dependent kinase inhibitor 2A
Co‐IP: co‐immunoprecipitation
CRISPR/Cas9: clustered regularly interspaced short palindromic repeats/CRISPR‐associated protein 9
DEG: differentially expressed gene
DMEM: Dulbecco's modified eagle medium
DRY (motif): aspartic acid‐arginine‐tyrosine (motif)
EGFR: epidermal growth factor receptor
ERK1/2: extracellular signal‐regulated kinase 1/2
FACS: fluorescence‐activated cell sorting
FBS: fetal bovine serum
GPCR: G‐protein coupled receptor
H&E: hematoxylin and eosin
HA (tag): Hemagglutinin (tag)
HNSCC: head and neck squamous cell carcinoma
HRP: Horseradish Peroxidase
KO: knockout
LSL: Lox‐STOP‐Lox
MAPK: mitogen‐activated protein kinase
mKT: mouse keratinocytes
MS: mass spectrometry
NETs: neuroendocrine tumors
NF‐κB: nuclear factor kappa‐light‐chain‐enhancer of activated B cells
ORF: open reading frame
PCR: polymerase chain reaction
PI3K: phosphoinositide 3‐Kinase
PIK3CA: Phosphatidylinositol‐4,5‐bisphosphate 3‐kinase catalytic subunit alpha
PPI: protein–protein interaction
PVDF: polyvinylidene difluoride
RI‐MUHC: Research Institute of the McGill University Health Centre
SCC: squamous cell carcinoma
SFM: serum‐free media
sgNTC: single guide RNA, non‐targeting control
sgRNA: single guide RNA
SSTR (SSTR1–5): somatostatin receptor (subtypes 1–5)
SSTR4/Sstr4: Somatostatin Receptor 4 (human/mouse gene nomenclature)
STRING: Search Tool for the Retrieval of Interacting Genes/Proteins
TCGA: The Cancer Genome Atlas
TIDE: tracking of Indels by decomposition
TNF‐α: tumor necrosis factor alpha
TP53: tumor protein p53

## 1 Introduction

Head and neck squamous cell carcinoma (HNSCC) affects mucosal lining of the upper aerodigestive tract in the head and neck regions including pharynx, larynx, the tongue, oral cavity, nasal cavity, and paranasal cavity [1]. Cutaneous squamous cell carcinoma (cSCC) is a form of skin cancer originating from keratinocytes in the skin [2]. Whole genome sequencing of HNSCC and cSCC patient tumors has identified hundreds of mutations including frequent genetic alterations in *TP53*, *PIK3CA*, *CDKN2A*, and *NOTCH1* mutations [3, 4, 5, 6, 7, 8]. The remainder of the alterations are infrequent but recurrent mutations, the vast majority of which lack biological or clinical validation [9]. A recent *in vivo* CRISPR/Cas9 knockout screen of 484 infrequently mutated genes in mouse models of HNSCC identified several tumor suppressors. One of the identified tumor suppressors was somatostatin receptor 4 (*SSTR4*), which is mutated in 2–3% of HNSCC and up to 5% of cSCC patients (Fig. S1A,B) [10]. When correlating the Sstr4 expression levels and survival outcomes in HNSCC, we found that tumors exhibit increased mRNA expression levels of SSTRs, suggesting a possible compensatory tumor‐suppressive mechanism to counteract oncogenic signaling (Fig. S1C–E).

Somatostatin receptors (SSTRs) are G‐protein coupled receptors (GPCRs) that belong to the Class A Rhodopsin family, which are the largest class of GPCRs (87%) [11]. Several intracellular signaling cascades such as the cyclic AMP (cAMP) signaling, MAPK signaling, and PI3K signaling are modulated by SSTR activation through G‐protein trimer (*α*, *β*, *ϒ*) uncoupling [12, 13, 14]. The downstream effect of these signaling pathways results in regulating the active states of several transcription factors through phosphorylation that are cell context dependent [15, 16, 17]. There are five subtypes of somatostatin receptors, SSTR1‐5, all of which are expressed ubiquitously throughout the human body [18, 19, 20]. SSTR1 and SSTR2 are highly expressed not only in the central nervous system (CNS) but also in the parotid glands, gastrointestinal (GI) mucosa, and glucagon/insulin‐secreting pancreatic cells [19]. SSTR4 demonstrates the greatest anatomical specificity, with its highest expression in the CNS, though not significantly higher than that of SSTR1 and SSTR2 [19, 20]. SSTR4 remains an understudied transmembrane protein with regard to cancer disease relevance, anatomical biodistribution and importantly its function in skin epithelial cells.

SSTR4 activation occurs through binding of endogenous physiological hormones such as somatostatin‐14 (SS‐14) or somatostatin‐28 (SS‐28) [21]. To reduce growth hormone production in primary adenomas and neuroendocrine tumors (NETs), FDA‐approved SSTR agonists such as octreotide has been particularly fundamental in the treatment of these cancers [12, 22, 23]. Small‐molecule synthetic SSTR4 agonists such as L‐803087 were synthesized decades ago but have only recently been shown to have functional effects in regulating cell proliferation and metastasis in cancer cell lines [24, 25]. J‐2156 is another synthetic agonist that displays nanomolar affinity to SSTR4 and to date has only been recorded in alleviating pain in rat and mouse models [26, 27].

In this study, using CRISPR/Cas9 gene editing, we generated relevant Sstr4 mouse keratinocyte cell lines and tested their phenotype by cell proliferation and flow cytometry assays. We utilized transcriptomics in addition to proteomic approaches to identify potential signaling pathways and protein–protein interactions (PPI) that can shed more light on the involvement of Sstr4 in HNSCC/cSCC. Through immunoassays, we found that Sstr4 is implicated in PI3K and MAPK signaling pathways. Importantly, through our immune‐competent *in‐vivo* mouse model, Sstr4‐knockout (Sstr4‐KO) and activation of Pik3ca^H1047R^ are sufficient for HNSCC/cSCC tumorigenesis and result in hyperplasia at 8 weeks of age. This study is the first to establish Sstr4 as a tumor suppressor in HNSCC/cSCC and delineates some of the intracellular mechanisms that govern these tumorigeneses.

## 2 Materials and methods

### 2.1 Animals

All experiments involving mouse models were done in the animal facility maintained at Research Institute of McGill University Health Center (RI‐MUHC). Ethical handling of mice and all experiments were conducted following approved standard operating protocol (AUP‐8224) in accordance with animal welfare and Canadian Council on Animal Care (CCAC) guidelines (CCAC, 2009). Equal numbers of male and female animals were used throughout the study without any bias. The animals used in this study were R26‐LSL (loxP‐STOP‐loxP) Pik3ca^H1047R/+^ mice (Gt(ROSA)26Sor^tm1(Pik3ca^^*^^H1047R)Egan^ in a clean FVBN background kindly provided by Egan S, SickKids) [28] and R26‐LSL‐Cas9‐GFP (#026175 in C57/Bl6 background) from Jackson Laboratories (Bar Harbor, ME, USA) [29]. Genotyping was performed by PCR using genomic DNA prepared from mouse ear punches [30]. Experimental mice were monitored once a week for tumor development and mice with tumors in their oral cavity, skin and head & neck regions were monitored every other day for their ability to eat/drink and weighed twice a week to monitor weight loss. When total tumor mass per animal exceeded 1000 mm^3^, mice were monitored biweekly and sacrificed.

### 2.2 Plasmid constructs

The lentiCRISPR v2 plasmid backbone (Addgene #52961, Watertown, MA, USA) [31] was used to establish the Sstr4‐KO sgRNA1 cell line. The plasmid backbone used for the *in vivo* mouse injection was pLKO‐NLS‐iCre stuffer v4 (Addgene #158032) which contained Cre‐recombinase [10]. Both plasmids had the same cloning strategy for inserting sgRNAs into filler regions of the plasmid. The lentiCRISPR v2 plasmid was digested with Esp3I to remove the 1885‐bp sgRNA filler. The forward and reverse oligos of each pair of sgRNAs were ordered as single‐stranded oligos, annealed together with T4 PNK and ligated into the vector. The guide sequences were verified with Sanger sequencing.

To construct the transgene expression plasmids with Sstr4‐DRY and Sstr4‐ORF constructs (Table S1), the gene blocks synthesized by TWIST Bioscience (South San Francisco, CA, USA) were amplified by PCR using TWIST Adapters. The LentiCas9 Blast plasmid (#52962; Addgene) [31] was digested with NheI and BamHI and the amplified TWIST fragments of Sstr4 gene blocks were also digested with NheI/BamHI and ligated to the plasmid. Successful cloning was confirmed by Sanger sequencing.

### 2.3 Lentivirus production and transduction

For low‐titer lentivirus production, HEK293T cells were cotransfected with the lentivirus packaging plasmids psPAX2 (#12260; Addgene), pMD2.G (#12259; Addgene), and transfer plasmid (e.g., Sstr4‐KO sgRNA1), which were mixed at a picomolar (pmol) ratio of 0.169 : 0.0936 : 0.2132 in serum‐free media (SFM). Polyethyleneimine (#23966; Polysciences, Warrington, PA, USA) was used as a transfection agent, and 1 : 3 DNA to PEI ratio was used. The following day, the SFM was changed to complete DMEM containing 10% FBS and 1% Pen/Strep. After 48–72 h, the lentiviral supernatant was collected and filtered through a 0.45 μm PES filter into 1.5‐mL centrifuge tubes before being stored at −80 °C until further use. For high titer lentiviral production, 150 mL of viral supernatant was collected and filtered through Stericup‐HV PVDF 0.45‐μm filter and then concentrated ~ 2000‐fold using the The Optima™ XPN ultracentrifuge. Viral titers were determined by infecting the R26‐LSL‐tdTomato primary keratinocytes and FACS based quantification.

### 2.4 Tracking of indels by decomposition (TIDE) analysis

To detect indels and quantify gene editing knockout (KO) efficiency we utilized TIDE analysis. Total Genomic DNA was extracted from transduced and puromycin selected primary mouse keratinocytes with Cas9 (KT‐CHEN) cell lines with each of the five Sstr4‐KO sgRNAs as well as wild‐type cell lines. Approximately 250‐bp region upstream and downstream of the expected indel site was amplified by PCR using primers specific for each sgRNA location called ‘TIDE primers’, which are listed in the Table S1. Sanger Sequencing was used to sequence the PCR products, and the editing efficiency was determined using https://tide.nki.nl.

### 2.5 Western blot

All protein lysates were denatured in 2X SDS Sample Buffer (Laemmli buffer) and boiled at 65 °C for 5 min. 10–20 μg of denatured protein lysates was resolved on a 10% polyacrylamide gel and transferred onto a 0.45 μm PVDF membrane. All washing was done with 1X TBST (0.5% Tween‐20 in Tris‐buffered Saline). Membranes were blocked with 5% non‐fat skim milk powder in TBST for 1 h followed by overnight 4 °C incubation with primary antibodies. The following primary antibodies were used: mouse Anti‐HA (#901514; BioLegend, San Diego, CA, USA), mouse Beta‐Tubulin HRP (#5346; Cell Signaling Technologies), rabbit Phospho‐ERK1/2 (#4370; Cell Signaling, Danvers, MA, USA), and rabbit ERK1/2 (#4695; Cell Signaling) primary antibodies at 1 : 4000 dilution. All secondary antibodies were either goat anti‐rabbit HRP or goal anti‐mouse HRP (#7074/#7076; Cell Signaling) used at 1 : 6000 dilution and incubated for 1 h at room temperature. The blot was incubated in Clarity Western ECL Substrate (#1705061; BioRad, Mississauga, ON, Canada) before imaging.

### 2.6 RNA‐sequencing and analysis

The Sstr4 cell lines KT‐CHEN, Sstr4‐KO, and SStr4‐ORF were cultured in 10‐cm plates. Only Sstr4‐KO and Sstr4‐ORF cell lines were incubated with 1 μm of J‐2156 agonist for 24 h at 37 °C and 5% CO_2_. After incubation, the cell culture plates were scraped, RNA was extracted and purified using Qiagen (Toronto, ON, Canada) RNeasy Plus Mini Kit (Cat. No. 74134). The purified RNA samples were library prepared and subsequently underwent Illumina Sequencing. RNA‐sequencing of each cell line was performed in duplicates. Briefly, the RNA samples first went quality control (QC) through sample quantitation, integrity, and purity using the Agilent 5400 Fragment analyzer system. The RNA Integrity Number (RIN) for all samples measured from Fragment analyzer were above 9.7. After QC analysis, library preparation was done by first purifying only messenger RNAs (mRNAs) through oligo poly‐T magnetic bead‐based separation. Between 20–25 million raw reads were obtained from Sstr4 cell lines and KT‐CHEN cell line. For bioinformatic analysis, the reads obtained underwent quality control using FastQC. Mapping and aligning of the reads to the reference mm10 mouse genome was done using STAR. Differential gene analysis was done using the DESeq2 R package by comparing read counts between KT‐CHEN and each Sstr4 sample. After identification of DEGs for each Sstr4 cell line, the DEGs underwent functional analysis on EnrichR by inputting the upregulated and downregulated DEG sets.

### 2.7 Long‐read sequencing of mouse tumors

Genomic DNA from tumor cells was isolated using the Wizard® Genomic DNA Purification Kit (Cat. No. A1120; Promega, Madison, WI, USA). 1 μg genomic DNA of the tumor was used as PCR template with FW (5′‐ CTGCTCTCCACCGCATTCC ‐3′) and RV (5′‐ CTGAAAGGGCCTGGCTTAGT −3′) primers for 35 cycles using the Hot Start Q5 2X Master Mix following the manufacturer's instructions (#M0496S; NEB, Whitby, ON, Canada). PCR products were resolved on a 1% agarose gel, and a clean ~ 1.3‐kb band was isolated using Zymo Gel DNA Recovery Kit (Zymo research, Irvine, CA, USA) as per manufacturer instructions. Samples were sent for sequencing to Plasmidsaurus for Oxford Nanopore sequencing resulting in ~ 3000 full‐length reads.

### 2.8 Immunoprecipitation and mass spectrometry

The Sstr4‐ORF cell line was treated with 1 μm of J‐2156 agonist for 6 h before harvesting total protein lysate. The cells were scraped after addition of 500 μL of Pierce IP Lysis Buffer and kept on ice for 3 h (Cat. No. 87787; Thermofisher). The lysate was centrifuged at 13 500 g for 5 min. The supernatant consisting of total protein lysate underwent protein pulldown using the Pierce Anti‐HA magnetic beads assay kit (Cat. No. 88836; Thermofisher, Saint‐Laurent, QC, Canada). Afterwards, the beads were sent for mass spectrometry (MS) analysis to the Proteomics Core. For the Co‐immunoprecipitation with western blot, HEK 293 cells were transiently transfected with HA‐Sstr4 and empty control vectors. 48 h after transfection, cells were lysed and processed as described before. The beads and input lysate were processed for western blotting after washes and the blot was probed for anti‐14‐3‐3 pan antibody (#95422; Cell Signaling) and anti‐HA antibodies. Vinculin was used as a control.

### 2.9 Cell culture

Primary mouse keratinocytes were cultured as described previously [10], which includes DMEM/F12 media supplemented with Pen Strep and chelated FBS at a final calcium concentration of 50 μm at 37 °C and 5% CO_2_. HEK 293T cells were cultured in DMEM media supplemented with 10% FBS and 1% Pen Strep.

### 2.10 Cell proliferation assay: Incucyte S3

Approximately 1000 cells were seeded in each well of a 96‐well plate. For each specific Sstr4 cell line, a total of six replicates were done with and without J‐2156 agonist to account for potential edge effect caused by media evaporation. The 96‐well plate with the seeded cells was inserted into the Incucyte S3 machine (Sartorius) where images of each well were captured every 6‐h interval until 66‐h time point where confluency was saturated. Plots were created using r studio and graphpad prism 10.3.1.

### 2.11 Cell cycle analysis

For cell cycle synchronization, cells at 90% confluency were serum starved for 24 h. To induce cell cycle, cells were passaged in complete media containing 10% FBS for 30 h. For propidium iodide (PI) staining, fixed cells were centrifuged at 1000 g and washed 3× with D‐PBS. 200 μg·mL^−1^ RNAase was added to 200 000 cells for 1 h at RT, then 100 μg·mL^−1^ PI was added. Cells were visualized by the BDFACS Fortessa. Cell cycle analysis was done by the flowjo software. The values of each cell cycle phase for each Sstr4 cell line were exported to prism 10.3.1 (graphpad) to visualize the G1, S, and G2 phases.

### 2.12 Statistics and reproducibility

All quantitative data are expressed as the mean ± SD. Differences between groups were calculated using several statistical tests such as Wilcoxon Matched‐paired signed rank test, 2‐Way ANOVA using Dunnet's Multiple Comparison Test, Linear Regression modeling, and Log‐Rank (Mantel‐Cox) statistical test using prism 10.3.1 (graphpad software). *P* < 0.05 denotes significance. All figures for schematics were made using BioRender.com website with license #GJ29XE6SN5.

## 3 Results

### 3.1 Generation and validation of Sstr4 knockout and overexpression keratinocyte cell lines

**Fig. 1:**
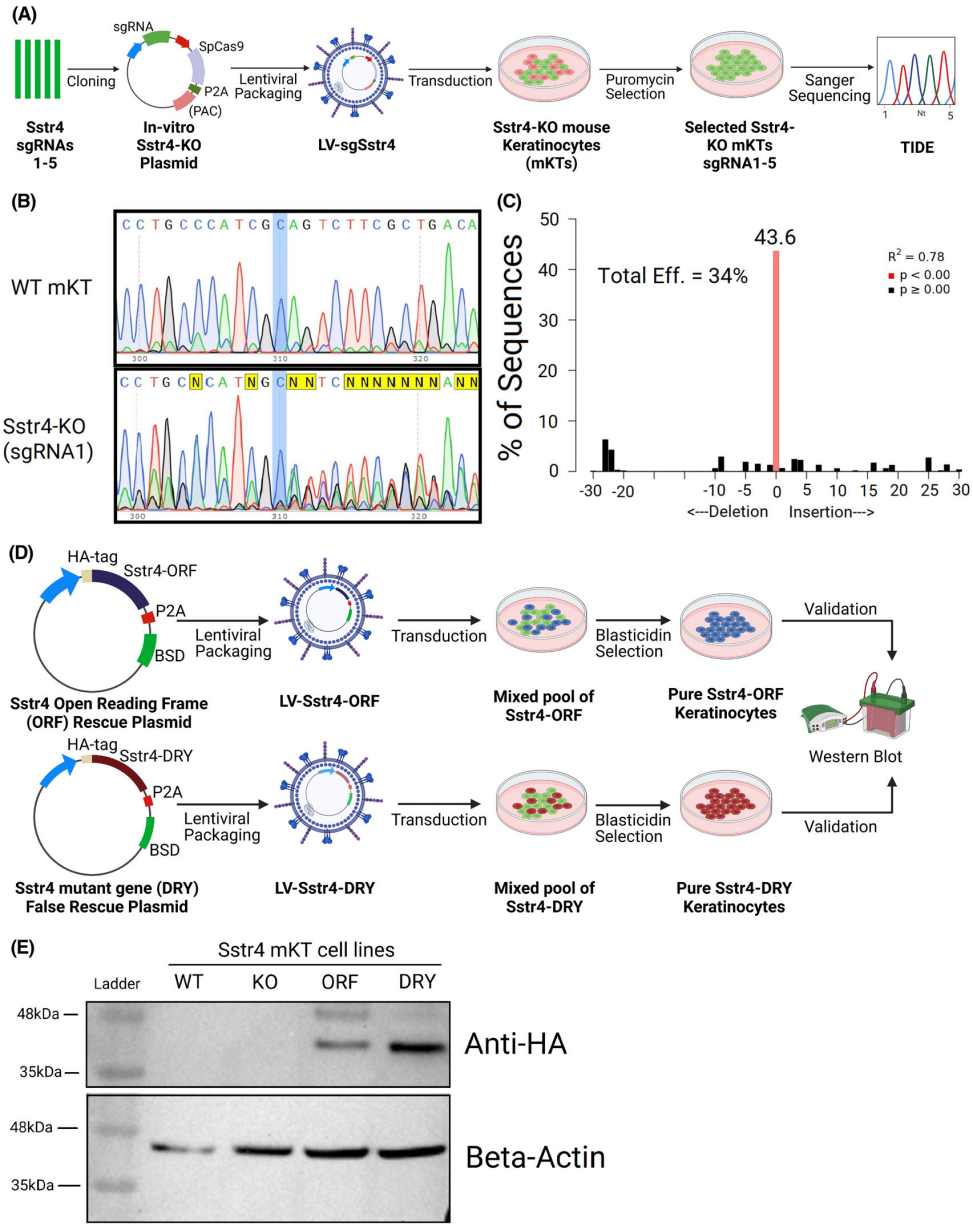
Generation and validation of genetically engineered Sstr4‐modified keratinocyte cell lines. (A) Schematic overview of the strategy used to generate Sstr4‐modified primary mouse keratinocyte cell line: CRISPR/Cas9‐mediated genome editing was performed using sgRNAs targeting Sstr4. (B) Representative chromatogram of wild‐type and Sstr4‐KO keratinocyte cell line after sanger sequencing: sgRNA1 exhibited high degree of chromatogram disruption, indicative of successful editing (*n* = 1). (C) Quantification of indel efficiency for sgRNA1 using TIDE analysis: sgRNA1 displayed an editing efficiency of approximately 34% and was selected for generation of the Sstr4‐KO cell line (*n* = 1). (D) Schematic overview of the strategy to generate wild‐type rescue (Sstr4‐ORF) and false rescue (Sstr4‐DRY) cell lines: Sstr4‐ORF represents over‐expression of wild‐type Sstr4 protein in the Sstr4‐KO parental background. Sstr4‐DRY represents overexpression of Sstr4 protein harboring point mutations D140G, R141G, and Y142G within the DRY motif in the Sstr4‐KO background. (E) Western blot analysis of HA‐tagged constructs expressed in Sstr4‐ORF and Sstr4‐DRY cell lines. Stable expression of each transgene was validated using anti‐HA antibody (1 : 4000), confirming successful lentiviral integration and protein expression (*n* = 1). Raw blots provided in Fig. S2.

To elucidate the mechanistic role of Sstr4 in skin keratinocyte cells, we first established a panel of genetically engineered primary mouse keratinocyte cell lines using CRISPR/Cas9 and lentiviral transduction. To generate a Sstr4 knockout (Sstr4‐KO) cell line, we tested five distinct single guide RNAs (sgRNAs) targeting Sstr4 (Table S1) to evaluate editing efficiency (Fig. 1A). Knockout efficiency for each sgRNA was assessed using Tracking of Indels by Decomposition (TIDE) analysis. We chose sgRNA1 cell line for downstream experiments, as it was previously shown to cause tumors in mouse models of HNSCC [10] and in our analysis, yielded the highest proportion of chromatogram aberration according to sanger sequencing and displayed an indel efficiency of 34% according to TIDE (Fig. 1B,C). The resulting cell line was designated as Sstr4‐KO and was used as parental cell line for generating rescue models. To functionally validate the role of Sstr4, we reintroduced a codon optimized wild‐type open reading frame (Sstr4‐ORF) into the Sstr4‐KO parental cell line, thereby creating a rescue condition. In parallel, we generated a mutant cell line, termed Sstr4‐DRY, by expressing an Sstr4 construct harboring triple point mutations (D140G, R141G, and Y142G) within the conserved intracellular DRY motif, which is critical for GPCR signaling (Fig. 1D). Expression of HA‐tagged constructs in all edited lines was confirmed by western blotting using anti‐HA antibodies, validating successful transgene integration and stable protein expression (Fig. 1E and Fig. S2).

### 3.2 Sstr4 plays a role in cell proliferation through G‐protein dependent and independent mechanisms

**Fig. 2:**
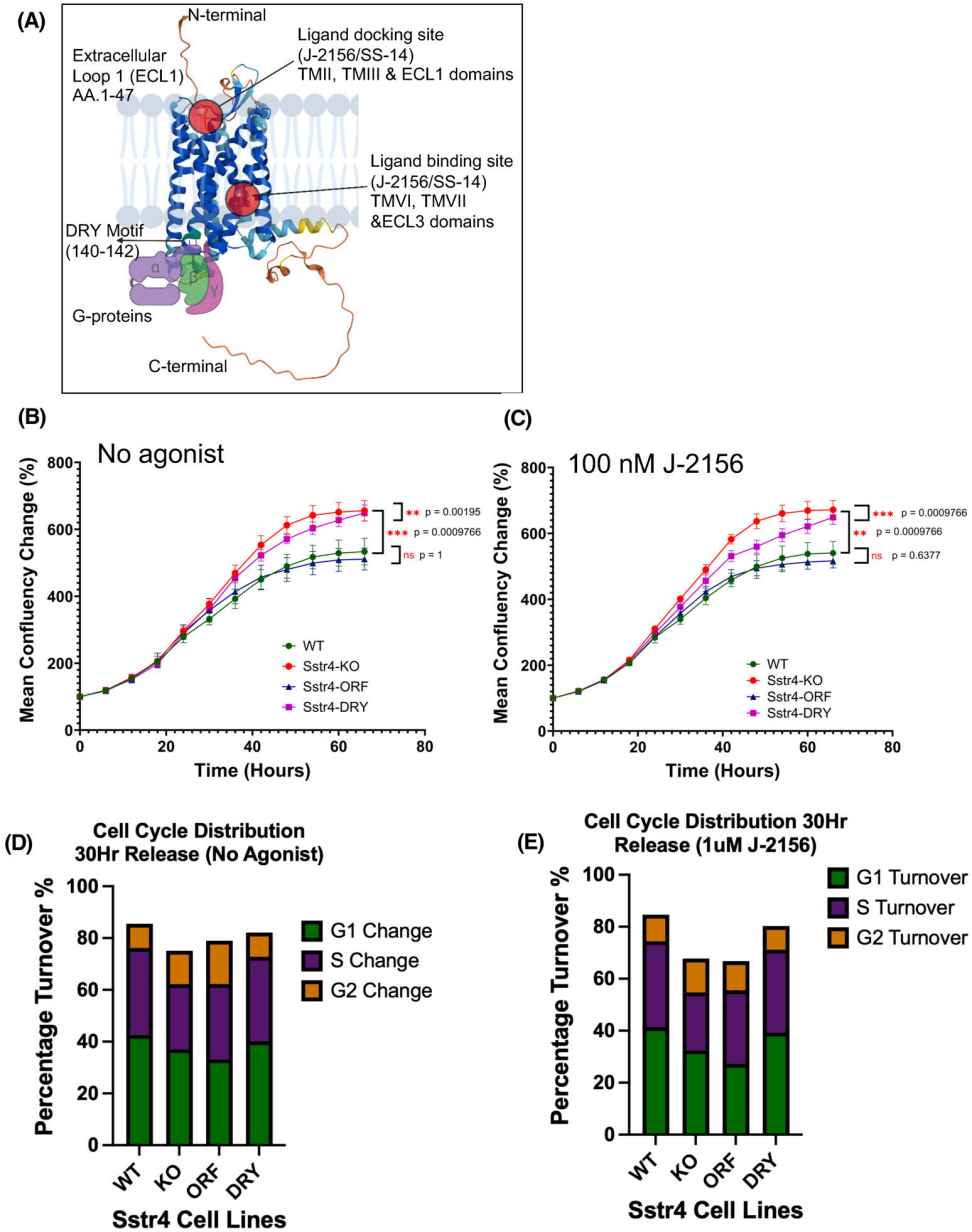
Incucyte S3 Real‐Time Cell Analysis and Flow Cytometry reveals cell proliferation effects of Sstr4 in Keratinocytes. (A) Crystal structure of mouse Sstr4 localized to the cell plasma membrane (AlphaFoldDB: P49660). The G‐proteins (Gα, Gβ, Gϒ) are associated with the intracellular side of Sstr4 and are shown as purple, green, and magenta colors. Sstr4 ligands such as J‐2156 agonist or somatostatin (SS‐14) are shown as a red sphere. (Created with BioRender.com). (B) Real‐time cell proliferation analysis using Incucyte S3 (Without Agonist). Cell lines expressing functional Sstr4 (WT and Sstr4‐ORF) showed significantly reduced proliferation compared to Sstr4‐deficient lines (Sstr4‐KO and Sstr4‐DRY). Data are shown as mean confluency over time from six replicates. Statistical analysis was performed using the Wilcoxon matched‐pairs signed rank test (*P* < 0.05). Linear regression analysis yielded *R* ^2^ > 0.93 and *P* < 0.0005 for comparisons between WT and Sstr4‐KO with and without agonist. (C) Real‐time cell proliferation analysis using Incucyte S3 (With Agonist). Cell lines expressing functional Sstr4 (WT and Sstr4‐ORF) showed significantly reduced proliferation compared to Sstr4‐deficient lines (Sstr4‐KO and Sstr4‐DRY). The effect of Agonist did not alter most cell lines except Sstr4‐DRY with a slight decrease. Data are shown as mean confluency over time from six technical replicates. (D, E) Flow cytometry analysis quantifying G1 phase turnover over a 30‐h period in the absence (D) and presence (E) of J‐2156 agonist. Cells were synchronized in G1 phase and released into complete media for 30‐h without agonist or with 1 μm J‐2156; No large differences were observed (*n* = 2). Statistical test using 2‐way Analysis of Variance (ANOVA) Dunnett's multiple comparison test was used and all error bars are mean ± SD. * = *P* < 0.05, ** = *P* < 0.005, *** = *P* < 0.0005, n.s = not significant.

To elucidate the functional role of Sstr4, we performed real‐time cell analysis to measure cell proliferation differences between Sstr4‐WT, Sstr4‐KO, Sstr4‐ORF, and Sstr4‐DRY (D140G, R141G, and Y142G, Fig. 2A) cell lines. Primary mouse keratinocyte cell lines containing a functional Sstr4 gene showed slower cell growth rate compared to cell lines that did not contain a functional copy of Sstr4 gene (Fig. 2B). Upon activation of Sstr4 with synthetic agonist J‐2156 across all cell lines, we observed a slight decrease in cell proliferation rate for the Sstr4‐DRY cell line (Fig. 2C). Sstr4‐DRY potentially does not allow G‐protein trimer coupling to the cytoplasmic domain of Sstr4 (Fig. 2A), and it is quite possible that in keratinocytes, Sstr4 may initiate signaling through G‐protein independent mechanism via heterodimerization with other receptors. Previous research found that in breast cancer cell line, activation of Sstr4 resulted in G1 cell cycle arrest [25]. We hypothesized a similar effect in keratinocyte cells and conducted a flow cytometry assay to quantify cell cycle phases to assess the effect on G1 arrest. We designed a flow cytometry assay using a modified stathmokinetic approach that arrests all cell lines in G1 cell cycle phase followed by release in complete media for specified duration to quantify cell cycle progression [32]. Our flow cytometry data indicate that there are differences in cell cycle distribution within each cell line when treated with and without J‐2156 agonist. Quantifying for the G1 turnover (difference between 0 and 30 h), we observed differences between all Sstr4 relevant cell lines (Fig. 2D). 37.1% of cells in Sstr4‐KO exited out of G1 and 33.2% of cells in Sstr4‐ORF exited out of G1. With the addition of J‐2156 agonist, 32.5% of cells in Sstr4‐KO exited out of G1, and 27.3% of cells in Sstr4‐ORF exited out of G1 (Fig. 2E). However, no statistical significance was observed. The WT and Sstr4‐DRY cell lines remain unaffected with or without 1 μm J‐2156 agonist.

### 3.3 RNA‐sequencing reveals key biological processes and pathways involved in Sstr4 signaling in mouse keratinocytes

**Fig. 3:**
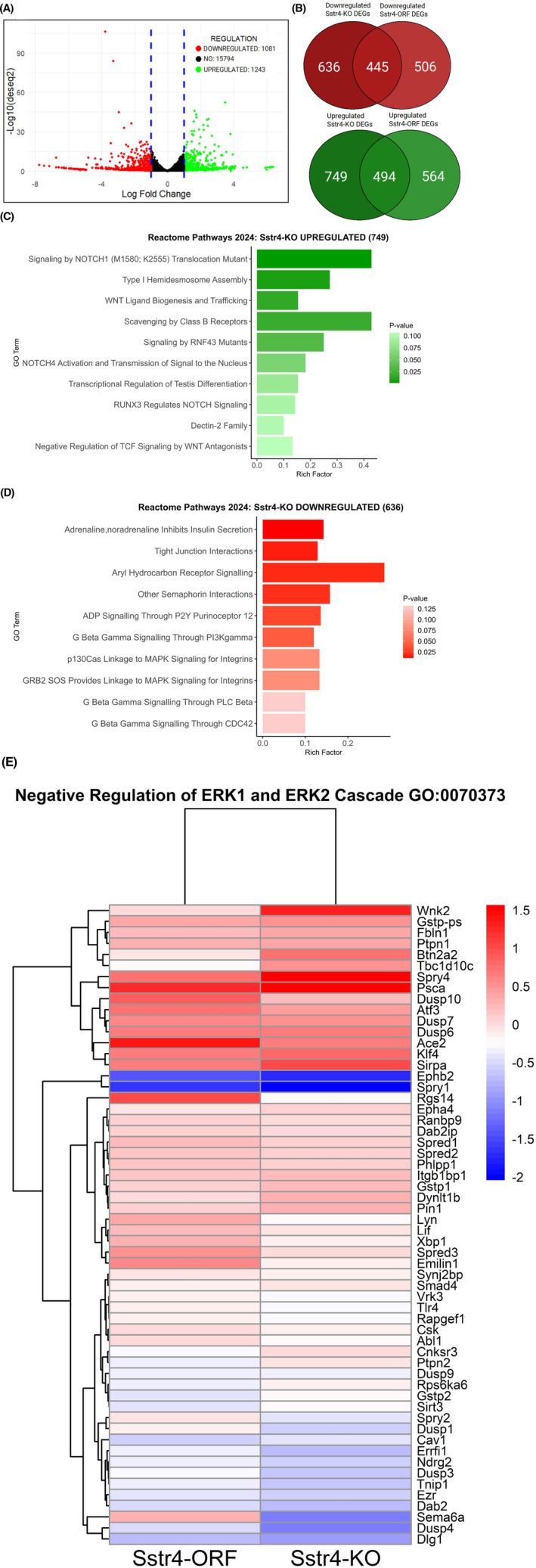
Transcriptomic profiling reveals Sstr4‐dependent signaling pathways in primary mouse keratinocytes. (A) Volcano Plot of Sstr4‐KO DEGs: Differential gene expression analysis between wild‐type (Sstr4‐ORF) and Sstr4 knockout (Sstr4‐KO) primary mouse keratinocytes. Volcano plot displaying significantly upregulated and downregulated genes in the Sstr4‐KO cell line. (B) Identification and removal of background noise genes from differential expression datasets: Approximately 500 genes commonly upregulated or downregulated between phenotypically opposing cell lines (Sstr4‐KO and Sstr4‐ORF) were excluded from downstream enrichment analysis to reduce nonspecific transcriptional noise. (C) Reactome Pathway Enrichment Analysis of 749 upregulated genes in Sstr4‐KO cell line. (D) Reactome Pathway Enrichment Analysis of 636 downregulated genes in Sstr4‐KO cell line. (E) Heatmap of DEGs between Sstr4‐KO and Sstr4‐ORF cell lines: Heatmap reveals prominent differences in the negative regulation of ERK1/ERK2 cascade, indicating restoration of MAPK signaling upon Sstr4 re‐expression.

To investigate the function and signaling of Sstr4 in keratinocytes, we performed transcriptomic profiling experiments on Sstr4‐ORF and Sstr4‐KO cell lines. Hundreds of genes were identified as differentially expressed genes (DEGs) between the two cell lines (Fig. 3A and Table S2). Before conducting pathway enrichment analysis, we removed approximately 500 upregulated and downregulated genes that were common between the two phenotypically opposite cell lines that contributed to background noise (Fig. 3B). Pathway enrichment of the unique upregulated genes identified in the Sstr4‐KO cells showed enrichment in pathways such as Wnt signaling, Notch signaling, and inflammation‐associated signaling that recruits immune cells involved in innate immunity (Fig. 3C). Conversely, the downregulated DEGs were enriched in pathways associated with PI3K/MAPK pathway, G‐protein trimer signaling, and Cdc42 GTPase activity (Fig. 3D). Comparing gene expression patterns between the Sstr4‐KO and Sstr4‐ORF cell lines, we observed most differences with regard to the negative regulation of ERK1 and ERK2 signaling cascade (Fig. 3E).

### 3.4 IP coupled mass spectrometry identifies novel protein–protein interactors (PPIs) of Sstr4

**Fig. 4:**
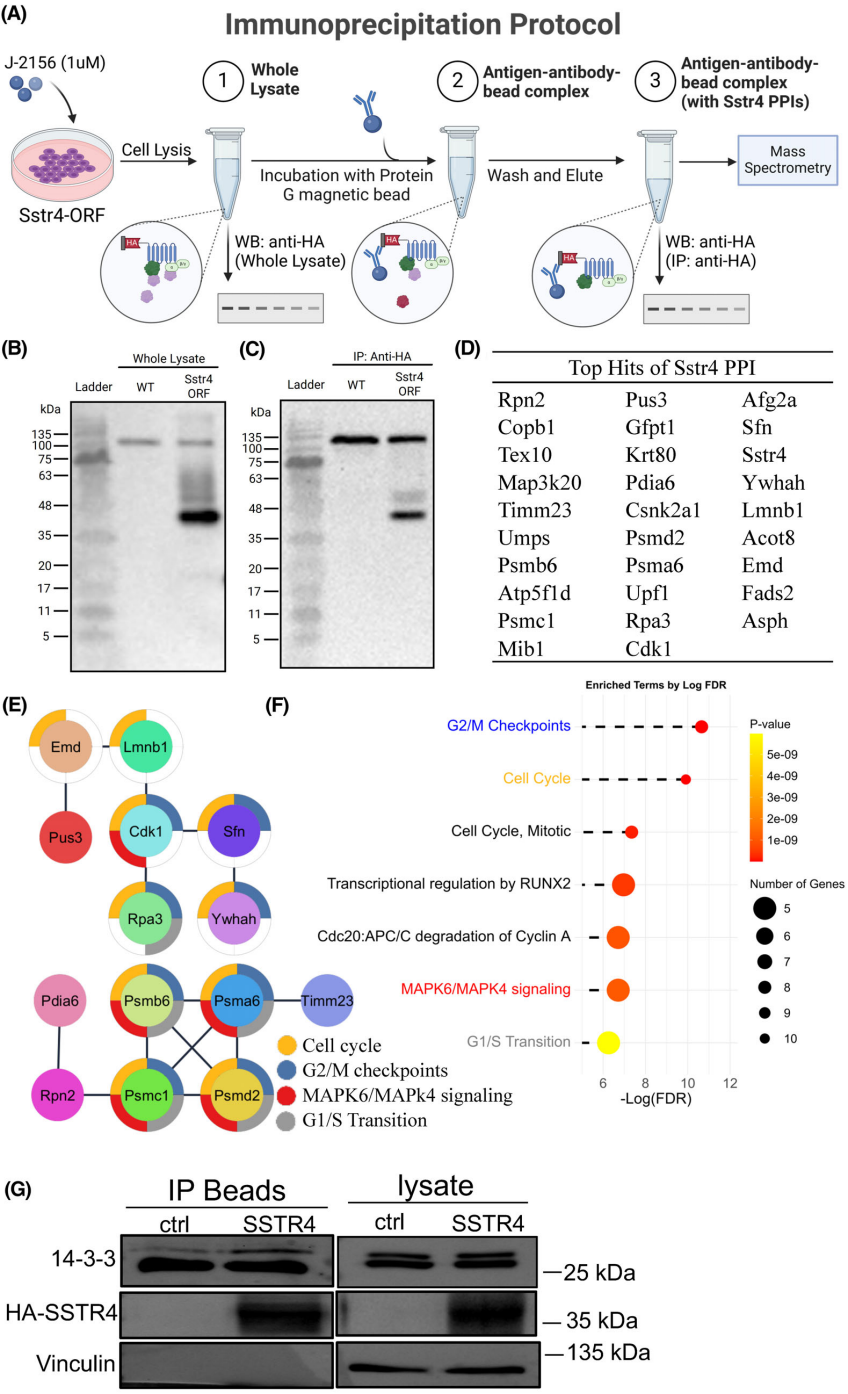
IP‐coupled mass spectrometry proteomics approach identifies novel protein–protein interactions (PPIs) of Sstr4 in keratinocytes. (A) Schematic of the co‐immunoprecipitation (Co‐IP) and mass spectrometry (MS) workflow. Cell lysates from HA‐tagged Sstr4‐ORF keratinocyte lines were incubated with anti‐HA magnetic beads to pull down interacting protein complexes. Eluted complexes were subjected to LC–MS/MS for identification of potential Sstr4 interactors. (B) Western blot analysis of whole cell lysates from the Sstr4‐ORF cell line prior to Co‐IP (*n* = 1). (C) Western blot validation of immunoprecipitated lysates confirming successful pulldown of Sstr4 and associated protein complexes from the Sstr4‐ORF cell line. (D) List of top protein interactors with Sstr4 in mouse keratinocytes as identified from IP‐Mass spectrometry (*n* = 2). (E) Protein–protein interaction network of Sstr4‐specific interactors identified from Co‐IP MS. STRING database analysis showed that 14 of these proteins form two discrete interaction networks, suggesting several coordinated and interconnected functional roles. Network made using cytoscape v3.10.3. Color around the circle corresponds to the legend. Raw data provided in Table S3. (F) Reactome pathway enrichment analysis: The 14 high‐confidence interactors forming the STRING network showed enrichment of pathways that are involved in cell cycle regulation and MAPK signaling. Font color corresponds to the specific protein in STRING network. (G) HEK293 cell lysates expressing HA‐Sstr4 or empty vector were subjected to immunoprecipitation (IP) with anti‐HA beads, followed by immunoblotting (IB) with anti‐14‐3‐3 (E9S9M) and anti‐HA antibodies. Input samples (5% total lysate) served as loading controls. The specific 14–3‐3 band (approx. 29 kDa) is enriched in the HA‐Sstr4 immunoprecipitate compared to the control (*n* = 2). Raw blots provided in Fig. S2.

Previous research conducted showed the heterodimerization capability between SSTR1/SSTR5 with EGFR through pbFRET assays [33]. Likewise, we aimed at pinpointing protein–protein interactions (PPIs) of Sstr4 and further exploring its heterodimerization capability in keratinocytes given that EGFR is persistently amplified in 38%–47% of HNSCC cases [8, 34]. To identify PPIs of Sstr4, we incubated cell lysate of Sstr4‐ORF (hemagglutinin‐HA‐tagged) with anti‐HA magnetic beads for co‐immunoprecipitation (Co‐IP) which was followed by mass spectrometry (MS) as depicted in the schematic (Fig. 4A). For quality control, during each step of the Co‐IP MS assay, we performed western blot of the lysates of the Sstr4‐ORF cell line (Fig. 4B,C). Mass spectrometry revealed 28 proteins that were unique interactors to Sstr4 with high statistical significance (Fig. 4D and Table S3), of which, 14 Sstr4‐associated proteins formed two discrete interaction networks, one consisting of six proteins and the other of eight proteins, suggesting the presence of functionally distinct protein complexes. (Fig. 4E). Our preliminary finding was the involvement of the scaffold protein 14‐3‐3*η* (Ywhah) as an interactor of Sstr4, which has also been shown to interact with other GPCRs and modulate GTPase activity of Rho/Rac family [35]. Reactome pathway analysis of these 14 proteins in STRING network revealed biological functions related to cell cycle and MAPK signaling (Fig. 4F). Co‐immunoprecipitation experiments revealed a physical interaction between HA‐tagged Sstr4 and endogenous 14‐3‐3. These results indicate that 14‐3‐3 proteins form a complex with Sstr4 (Fig. 4G).

### 3.5 Activation of Sstr4 by J‐2156 agonist results in phosphorylation of ERK1/2 involved in MAPK signaling

**Fig. 5:**
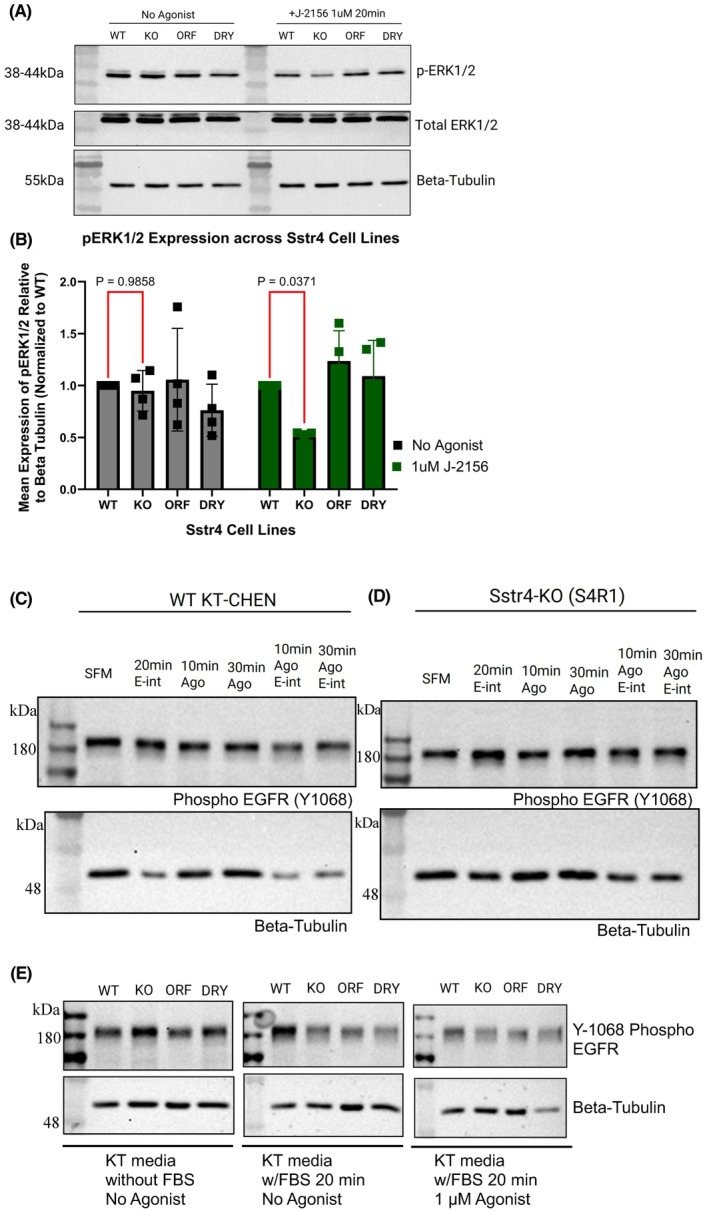
Western Blot indicating the effects on phospho‐ERK1/2 and phospho Y‐1068 EGFR upon activation of Sstr4 in keratinocytes. (A) Western blot analysis of phospho‐ERK1/2. All keratinocyte cell lines wild‐type (WT), Sstr4‐KO, Sstr4‐ORF, and Sstr4‐DRY cell lines were treated with or without J‐2156 agonist for 20 min before the lysates were analyzed by western blot for changes in levels of phospho‐ERK1/2 (*n* = 3). (B) Densitometric quantification of phospho‐ERK1/2. The levels of phospho‐ERK1/2 were quantified and normalized to beta‐tubulin. Significant differences in phospho‐ERK1/2 levels were observed between WT and Sstr4‐KO cells only upon J‐2156 treatment. Statistical test using 2‐way analysis of variance (ANOVA) Dunnett's multiple comparison test was used. Data are mean ± standard deviations of three technical replicates (*n* = 3); *P* < 0.05. (C) Western blot analysis of phospho‐EGFR (Y1068) levels under various agonist stimulation conditions in WT keratinocytes: No pattern in phosphorylation was detected. E‐int indicates the keratinocyte media with serum used and ago indicates the J‐2156 agonist (*n* = 1). (D) Western blot analysis of phospho‐EGFR (Y1068) levels under various agonist stimulation conditions in Sstr4‐KO keratinocytes. No definitive changes were observed (*n* = 2). (E) Western Blot analysis for phospho‐Y1068 EGFR across WT, KO, ORF, and DRY cell line conditions. The rate of phospho‐EGFR (Y1068) was quantified following media transition from serum‐free to complete medium, with or without J‐2156 agonist (*n* = 1). Raw blots provided in Fig. S2.

To further decipher the intracellular signaling mechanisms responsible for oncogenic signaling due to dysfunctional Sstr4, we investigated the effect of Sstr4 in MAPK signaling which both the transcriptomic and proteomic data predicted. Western blots for phospho‐SAPK/JNK, phospho‐p38 (not shown), and phospho‐ERK1/2 were performed. Only phospho‐ERK1/2 yielded positive data. Upon Sstr4 activation by the agonist, an increase ERK1/2 phosphorylation was observed across all cell lines except Sstr4‐KO (Fig. 5A). Furthermore, Sstr4‐DRY cells treated with agonist showed similar effects as in Sstr4‐WT and Sstr4‐ORF cells, which reinforces the possibility of G‐protein independent signaling mechanisms potentially through 14‐3‐3 proteins that do not require physical association to the DRY motif of Sstr4. Densitometric analysis revealed significant differences in phospho‐ERK1/2 between Sstr4‐WT and Sstr4‐KO cells when treated with J‐2156 agonist (Fig. 5B). The Y1068 and Y1086 tyrosine residues of EGFR are critical phosphorylation sites that facilitate MAPK/ERK pathway activation through Ras GTPase signaling. Phosphorylation at these sites is often associated with constitutive oncogenic signaling, particularly in cancers of epithelial origin [36]. Given that 14‐3‐3 proteins modulate the activity of various GTPases, including those upstream of MAPK signaling, these observations underscore the importance of further elucidating the Sstr4 signaling axis, particularly its potential crosstalk with EGFR tyrosine kinase activity in the context of HNSCC. Initial experiments testing a range of agonist stimulation conditions in Sstr4‐WT keratinocytes did not yield consistent changes in phospho‐EGFR (Y1068) levels (Fig. 5C). Similarly, analysis in Sstr4‐KO cells failed to show a definitive pattern of EGFR Y1068 phosphorylation (Fig. 5D). Upon revisiting the experimental design, we identified the presence of endogenous somatostatin in the culture media as a potential confounding factor, possibly masking ligand‐specific effects of Sstr4 activation. To address this, we redesigned the assay to include a time‐course analysis of phospho‐Y1068 EGFR following a media switch from serum‐free to complete medium, with or without J‐2156. Under these optimized conditions, we observed a decrease in phospho‐Y1068 EGFR levels upon J‐2156 stimulation across all keratinocyte cell lines (Fig. 5E), suggesting that Sstr4 activation could possibly exert its tumor suppressive function through negatively regulating EGFR signaling.

### 3.6 Sstr4 functions as a tumor suppressor in Pik3ca^H1047R^ oncogenic HNSCC/cSCC mouse model

**Fig. 6:**
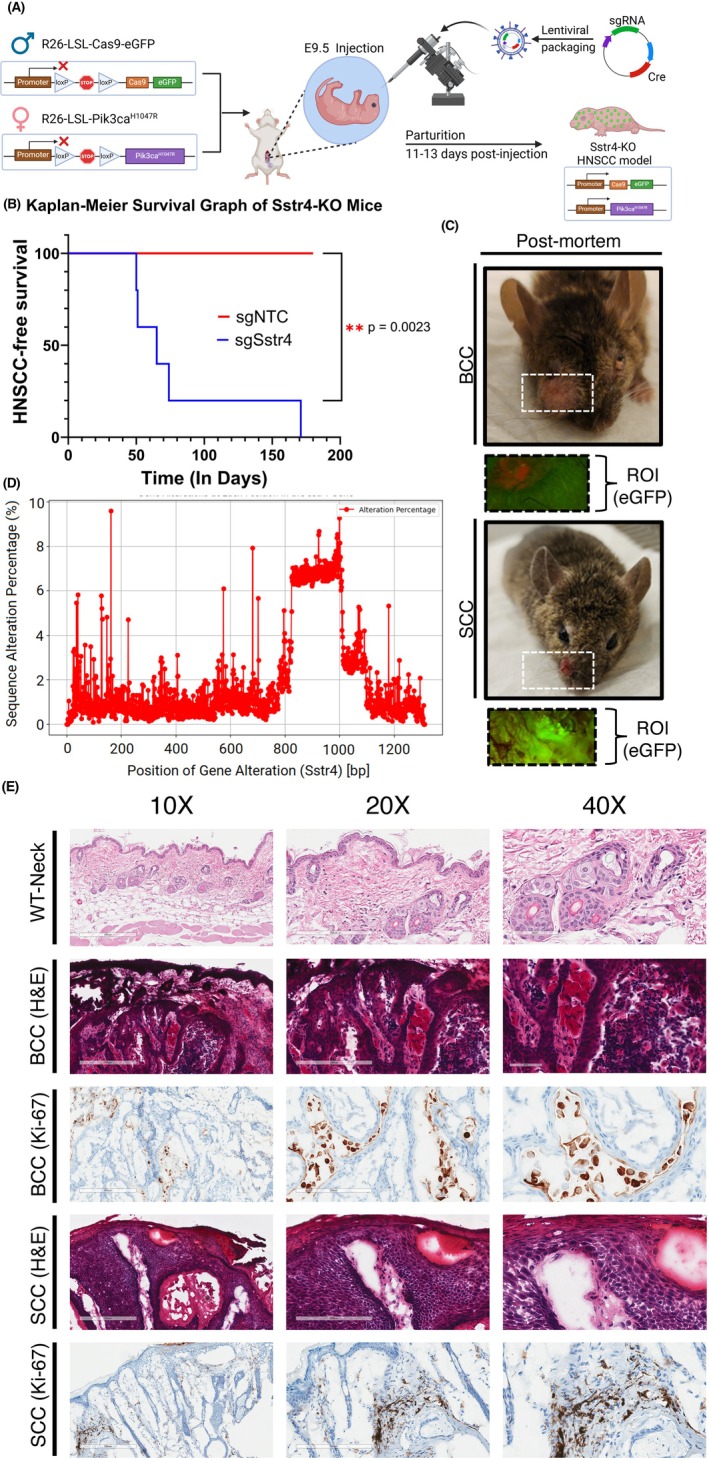
*In vivo* validation of Sstr4 as a tumor suppressor in an HNSCC mouse model using ultrasound‐guided in utero lentiviral delivery. (A) Schematic representation of the *in‐utero* ultrasound‐guided lentiviral injection. The procedure is used to induce targeted knockout of *Sstr4* in the basal epithelial layer of developing embryos carrying the Pik3ca^H1047R^ allele. The lentiviral packaged plasmid construct encodes Cre recombinase and sgRNAs targeting Sstr4 to activate Pik3ca^H1047R^, GFP and Cas9 for gene editing. (Created with BioRender.com). (B) Kaplan–Meier survival curve of Sstr4‐KO mice (*n* = 5) and sgNTC (*n* = 6). Sstr4‐KO (*n* = 5) also includes the two mice that did not form tumors but died due to unexplained mortality. NTC: Nontargeting control sgRNAs. Log‐Rank (Mantel‐Cox) statistical test done on graphpad prism. ** = *P* < 0.05. (C) Images showing two distinct tumor morphologies in Sstr4‐KO mice: basal cell carcinoma (BCC) and squamous cell carcinoma (SCC). GFP fluorescence confirmed clonal tumor origin due to successful lentiviral integration and Sstr4 knockout. (D) Deep sequencing of the Sstr4‐KO tumor: ~ 3000 full‐length reads obtained via Oxford Nanopore technology. Mutation frequency ranged from 2 to 8% per nucleotide position, with hotspot regions between nucleotides 800–1000. (E) Histopathological analysis of Sstr4‐KO tumors: Hematoxylin and eosin (H&E) staining revealed distinct pathology consistent with basal hyperplasia in BCC and suprabasal hyperplasia in SCC. Ki‐67 immunohistochemistry indicated active proliferation in both tumor types (Scale bar = 60 μm).

Given that Sstr4 was identified as a potential tumor suppressor from our previous study involving *in‐vivo* CRISPR‐Cas9 knockout screen of HNSCC long‐tail gene library, we aimed to validate its tumor suppressor role in HNSCC as well as cSCC tumorigenesis [10]. We sought to determine whether loss of Sstr4 coupled to activation of PI3K pathway through Pik3ca^H1047R^ mutant protein expression contributes to cancer initiation *in vivo*. To this end, we utilized the established HNSCC/cSCC mouse model, enabling targeted knockout of Sstr4 (Sstr4‐KO) precisely in basal epithelial cells of the stratum basale [10, 30]. The stratum basale is composed of keratinocyte stem cells that are responsible for skin renewal and upward cellular differentiation to form the stratum spinosum and stratum granulosum layers of epithelium, which are the cells of origin of HNSCC [37, 38, 39]. A schematic representation of this approach is shown in Fig. 6A. Following successful surgery and completion of parturition, a total of seven mouse pups were born (Sstr4‐KO experimental group). Of these, five mice survived to young adulthood, all of which were male. Among the surviving mice, three developed tumors (60% tumor incidence), as shown in Fig. 6B. The remaining two pups experienced unexplained mortality during the early postnatal period. The six mice injected with nontargeting control sgRNA (sgNTC) did not show any indication of tumorigenesis until experimental endpoint which indicates a 0% tumor incidence. Figure 6B recapitulating our previous phenotype [10, 30]. Interestingly, we observed the development of two phenotypically distinct tumors: squamous cell carcinoma (SCC) and basal cell carcinoma (BCC) (Fig. 6C). Visually, the tumors could be distinguished as SCC if they appeared scaly and reddish, or BCC if they appeared as bumps [40, 41]. Additionally, Cre recombinase activity in lenti‐virus transduced clones triggers expression of both Pik3Ca^H1047R^ and Cas9‐GFP across the tumor tissue indicating tumor clonality specifically due to Sstr4 knockout and activation of PI3K signaling (Fig. 6C).

To confirm the presence of Sstr4‐KO, we performed Oxford Nanopore sequencing of the DNA extracted from the mouse tumor, which gave us approximately ~ 3000 full‐length reads of Sstr4 gene. The frequency of gene alterations (insertions, mutations, deletions) for each position of the Sstr4 gene was between 2 and 8% (Fig. 6D). The frequency is expected to be lower as the tumor environment contains other cell types and immune infiltrates that have intact *Sstr4*. Highest gene alterations were between 800 and 1000 nt which correspond to sgRNA2 and sgRNA5 sequences that also showed high indel efficiency *in vitro*. Immunohistochemical staining with hematoxylin/eosin (H&E) and Ki‐67 (presence of actively proliferating cells) showed differences in tumor pathology between wild‐type and SCC/BCC tumor samples (Fig. 6E). BCC pathology was consistent with basal hyperplasia and SCC pathology was consistent with suprabasal hyperplasia.

Taken together, our data show that Sstr4 functions as a tumor suppressor in HNSCC/cSCC and possibly in BCC through modulation of MAPK/ERK signaling.

## 4 Discussion

Whole genome sequencing studies show that most adult solid cancers accumulate hundreds of somatic mutations—including classic driver mutations in TP53, PIK3CA, CDKN2A, RAS genes etc. Despite extensive cancer genome sequencing, the specific mutational combinations that drive cancer initiation, progression and treatment response remain elusive, highlighting the need for deeper mechanistic understanding to advance cancer biology and precision medicine. A recent *in vivo* functional genomics study utilizing mouse models of HNSCC identified *Sstr4* as one of the putative tumor suppressor genes that works in co‐operation with oncogenic Pik3ca^H1047R^ mutation.

The primary aim of this study was to investigate the role of Sstr4 in HNSCC/cSCC tumorigenesis. As indicated in our *in vivo* experiments utilizing the ultrasound‐guided in utero lentiviral injection, our preliminary results demonstrate that the loss of *Sstr4* coupled to the overexpression of Pik3ca^H1047R^ oncogenic mutant is sufficient to initiate tumorigenesis in our mouse model. To date, no literature has established the tumor suppressor role of Sstr4. This study is the first to show the tumorigenic role of Sstr4 in HNSCC/cSCC using an immune‐competent mouse model and a clonal loss‐of‐function in a native setting, within the intact structure of the organ. While our *in vivo* results are a preliminary verification of Sstr4 tumor suppressor function in the presence of Pik3ca^H1047R^, the small sample size (*n* = 5) and lack of gender balance limit the interpretation of tumor penetrance or sex‐specific effects. Larger sample size and gender‐balanced cohorts will be required for robust validation in the future.

An intriguing aspect of our *in vivo* data is the observed heterogeneity in cancer progression. Specifically, we identified the development of two distinct forms: basal cell carcinoma (BCC) like and squamous cell carcinoma (SCC) like in the skin, head and neck regions. SCC was observed at a later time point than BCC despite identical genetic manipulation of cells in the epidermal layers. One possible explanation for the observation is that the development of distinct forms of cancer depends on the cell origin. This suggests that the tumor suppressive function of Sstr4 is critical not only in the stratum spinosum/granulosum layers, but also in the stratum basale, all of which contain keratinocytes responsible for the formation of SCC and BCC, respectively [40, 41]. These findings emphasize potentially the highly localized, layer‐specific role of Sstr4 in HNSCC/cSCC as well as BCC tumorigenesis, but more detailed investigation is required to clearly understand how Sstr4 drives either of these cancers. In addition, further studies are required to understand the endogenous SSTR4 protein and gene expression levels in normal human keratinocytes versus HNSCC‐derived cell lines for translational relevance.

As SSTR4 is less studied compared to other somatostatin receptor family members, we aimed to decipher the intracellular signaling mechanisms by which Sstr4 exerts its tumor suppressor function in HNSCC/cSCC. The RNA‐sequencing data presented in this study provided key insights into the role of Sstr4 in mouse keratinocytes, particularly with respect to enrichment in several biological processes and pathways. One novel finding was the enrichment of several pathways previously not reported for Sstr4 in any cell line such as Wnt signaling, Notch signaling, S1PR1 signaling, AHR signaling, EGFR signaling, and FGF signaling [12]. Earlier reports have mentioned SSTR2 to be involved in Notch, NF‐κB, and TNF‐α pathways, whereas SSTR4 has not [12, 42, 43, 44]. Wnt/beta‐catenin pathway activation has not been studied as a result of SSTR activation; however, inhibition of Wnt decreased SSTR expression in neuroendocrine tumors (NETs) [22].

The proteomic data obtained from pulldown mass spectrometry (MS) found new PPIs for Sstr4. One major class of proteins is the 14‐3‐3 proteins, specifically the 14‐3‐3*η* (Ywhah). To date, no studies have shown 14‐3‐3 to be associated with SSTRs. While the Co‐IP pull down experiments validated this interaction, it will be interesting to understand further mechanisms behind this interaction. We theorize that these 14‐3‐3 proteins associate with GPCRs such as SSTR5 on the cytoplasmic side through the PDZ motif which Sstr4 also harbors [35]. The cell proliferation assay with the Sstr4‐DRY cell line and the J‐2156 agonist showed a slight reduction and significant difference in cell growth rate compared to Sstr4‐KO. This observation indicates that cell proliferation is controlled in a G‐protein‐independent manner through the 14‐3‐3 signal adaptor and scaffold proteins. Through a series of western blots, we identified that Sstr4 activation results in increased expression of phospho‐ERK1/2. This aligns with previous findings that MAPK signaling is involved in Sstr4 signaling [45, 46]. While our transcriptomic, proteomic, and phospho‐ERK data provide convergent molecular evidence supporting the Sstr4‐MAPK/ERK axis in cell lines, this regulatory relationship has not yet been definitively established *in vivo* and will require direct pathway perturbation experiments in future studies.

## 5 Conclusion

Our study establishes the role of SSTR4 as a tumor suppressor that co‐operates with oncogenic PIK3CA^H1047R^ mutation to drive HNSCC/cSCC and potentially BCC tumorigenesis. While transcriptomic and proteomic studies involving keratinocytes indicate the role of MAPK signaling, further research is required to pinpoint the precise mechanism of SSTR4 tumor‐suppressive mechanism.

## Conflict of interest

All authors declare no conflict of interest.

## Author contributions

AT had full access to all the data in the study and takes responsibility for the integrity of the data. SKL and AT were involved in concept and design. AT, TKH, AK, ZK, YY, AJL, and SKL were involved in acquisition, analysis, or interpretation of data. AT and SKL were involved in drafting of the manuscript. All authors were involved in critical revision of the manuscript for important intellectual content. AT was involved in statistical analysis. SKL obtained funding. SKL was involved in administrative, technical, or material support. SKL was involved in supervision.

## Supporting information


**Table S1.** List of (a) mouse Sstr4 sgRNAs (5 total), (b) codon optimized sequences for mouse WT Sstr4 and DRY motif mutant Sstr4 and (c) Primers used for tide analysis are listed.

**Table S2.** RNA‐seq data of Sstr4 KO, WT keratinocytes and Sstr4 ORF expressing keratinocytes.

**Table S3.** IP‐Mass spec data counts of control vs HA‐tagged Sstr4‐ORF expressing keratinocytes.

**Fig. S1.** Mutational frequency of SSTR4 in head and neck squamous cell carcinoma and cutaneous squamous cell carcinoma.
**Fig. S2.** Raw/unedited blots of western blots.

## Data Availability

All data generated or analyzed during this study are included in this article and its Supporting Information files. Further enquiries can be directed to the corresponding author.
