# Supplementary material for: Somatostatin receptor 4 (SSTR4) is a tumor suppressor in cutaneous and head & neck squamous cell carcinomas
Source: Mol Oncol. 2026 Aug 7:10.1002/1878-0261.70316. Online ahead of print. doi: 10.1002/1878-0261.70316 (PMC13451653; doi:10.1002/1878-0261.70316)
Supplement: Supplementary file 1 [file MOL2-9999-0-s002.pdf]

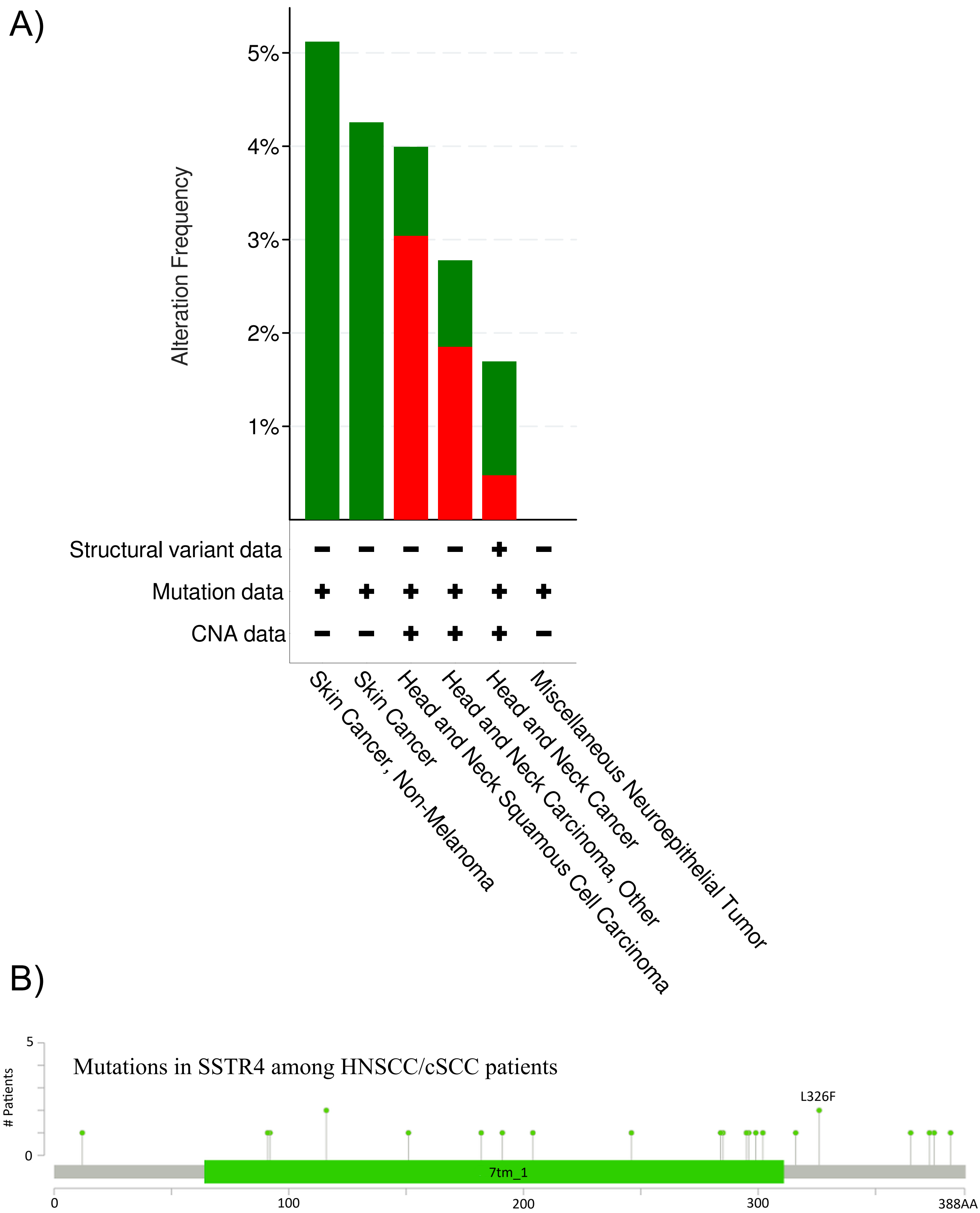
A)
5%
4%
3%
2%
1%
Alteration Frequency
Structural variant data
Mutation data
CNA data
Skin Cancer, Non-Melanoma
Skin Cancer
Head and Neck Squamous Cell Carcinoma
Head and Neck Carcinoma, Other
Head and Neck Cancer
Miscellaneous Neuroepithelial Tumor
B)
5
0
Patients
Mutations in SSTR4 among HNSCC/cSCC patients
L326F
7tm_1
0
100
200
300
388AA

C)

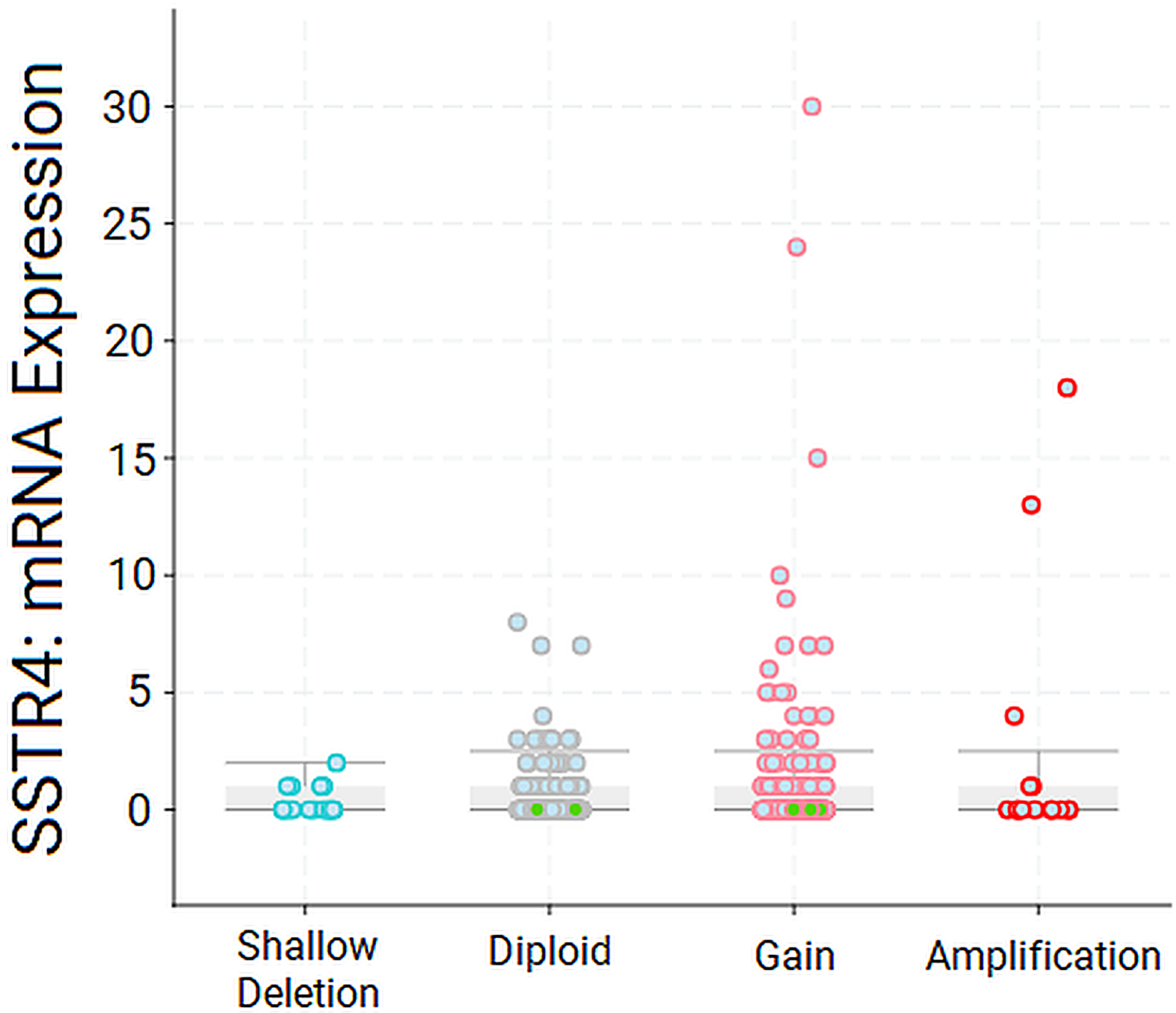
SSTR4: mRNA Expression
30
25
20
15
10
5
0
Shallow Deletion
Diploid
Gain
Amplification

D)

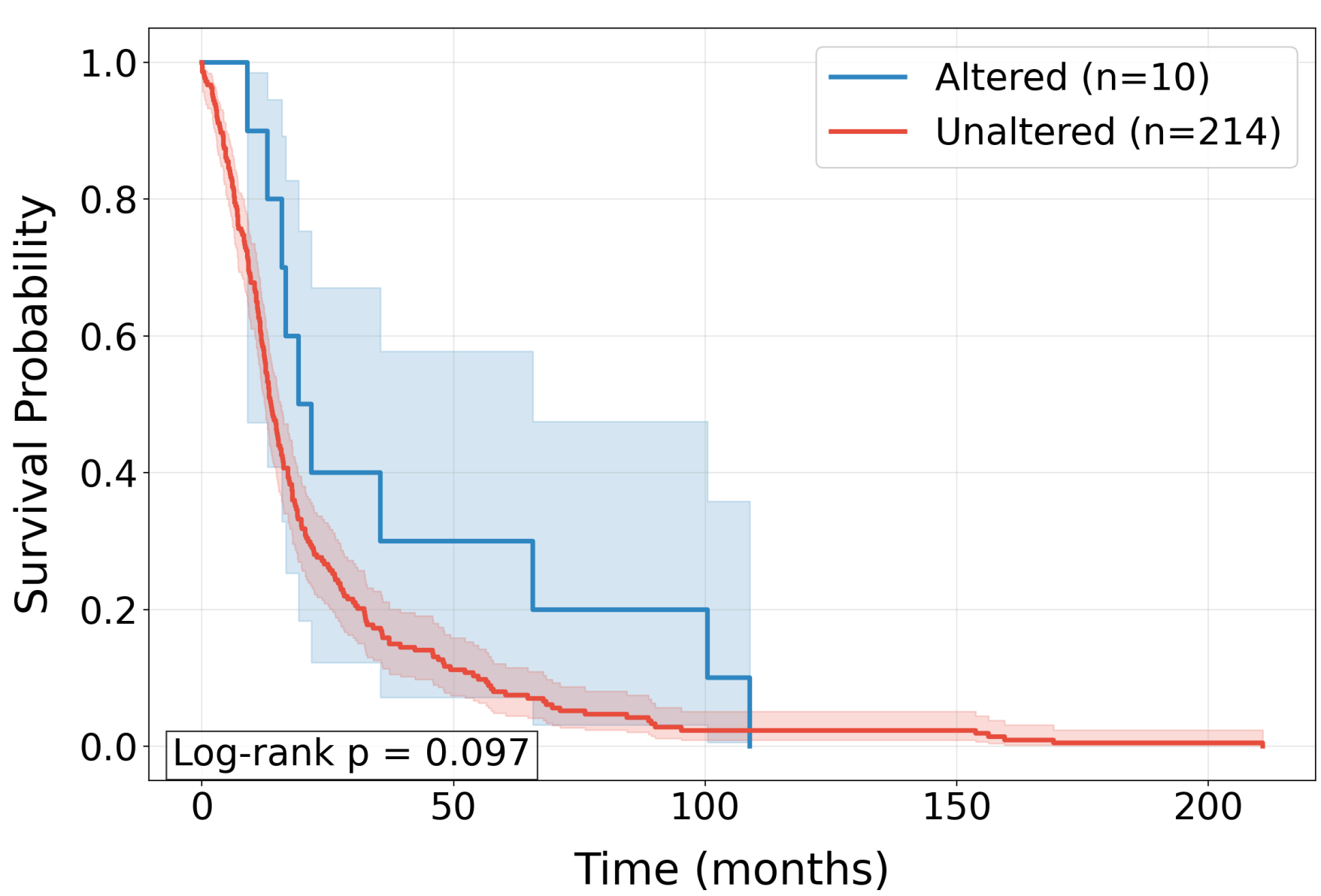
Altered (n=10)
Unaltered (n=214)
Survival Probability
1.0
0.8
0.6
0.4
0.2
0.0
Log-rank p = 0.097
0
50
100
150
200
Time (months)

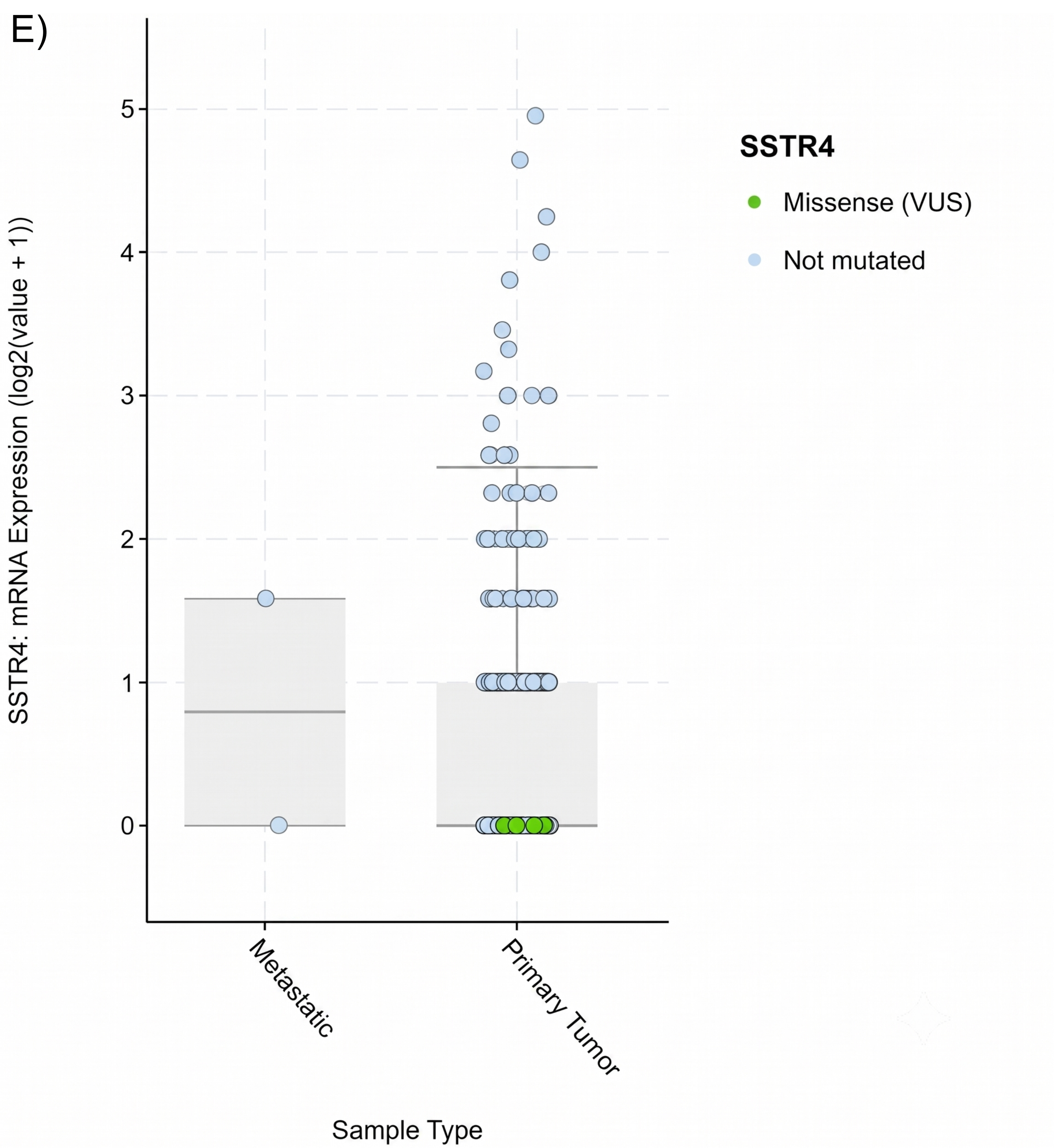

Supplementary Figure 1: A) Mutational frequency of SSTR4 in Head and Neck squamous cell carcinoma and cutaneous squamous cell carcinoma as obtained from cbioportal.org B) Location of all the missense mutations on SSTR4 protein identified from HNSCC/cSCC patients identified from cbioportal.org. C) mRNA expression of SSTR4 in patient samples from TCGA D) KM plot showing the survival probability of patients with altered SSTR4 expression compared to unaltered group. Please note the sample size is small for the altered group vs unaltered group. E) mRNA expression of SSTR4 in primary vs metastatic tumors. The mutations are indicated in circles.

Figure.1 E RAW Blots

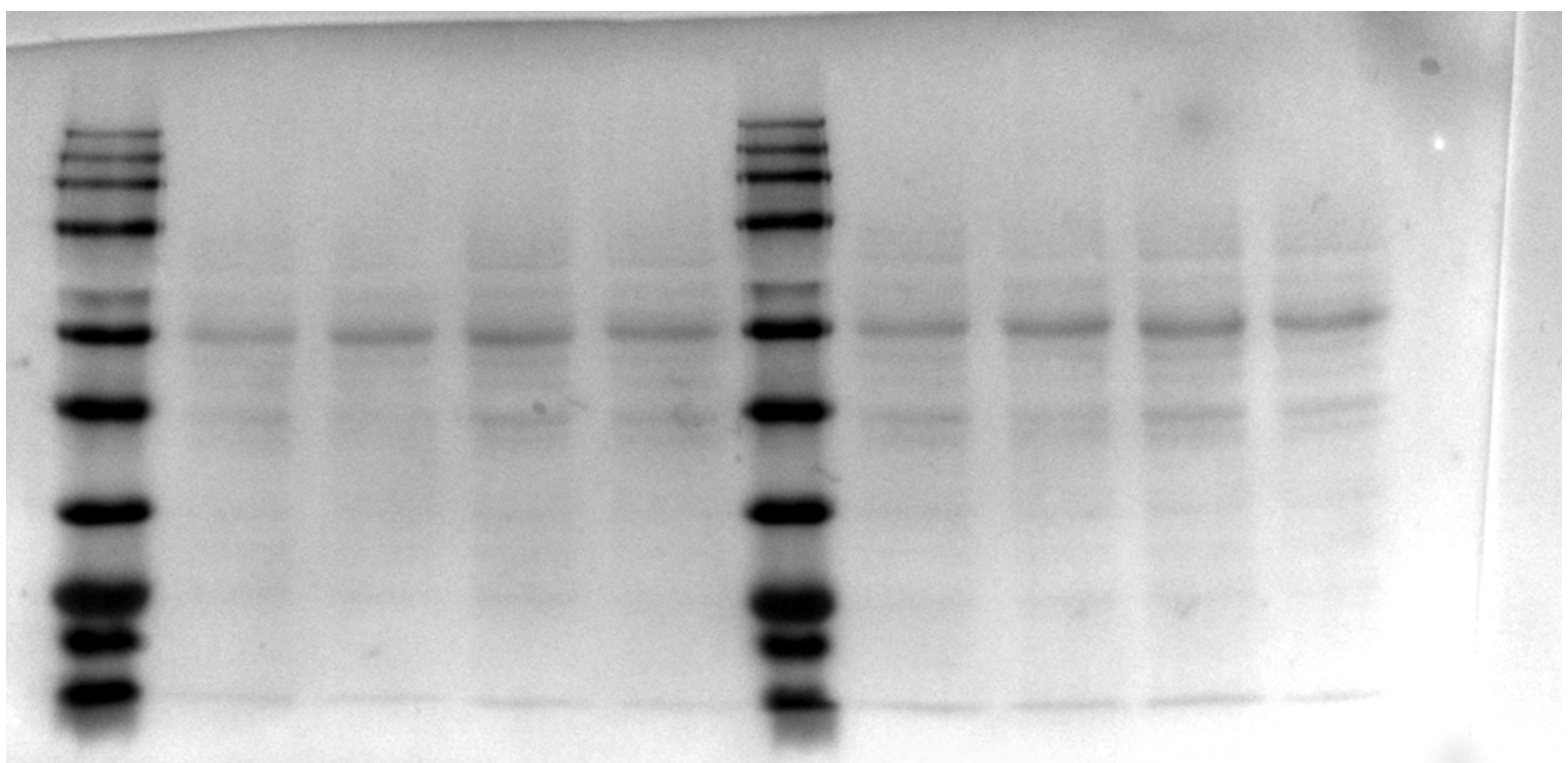

ponceau

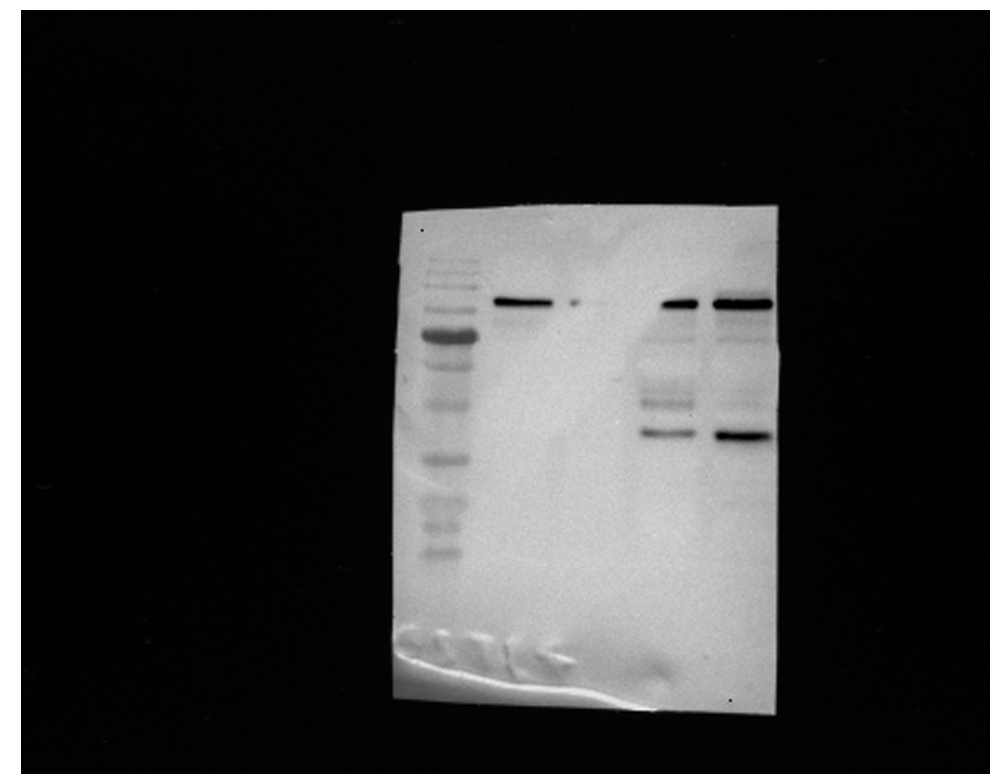

Anti-HA

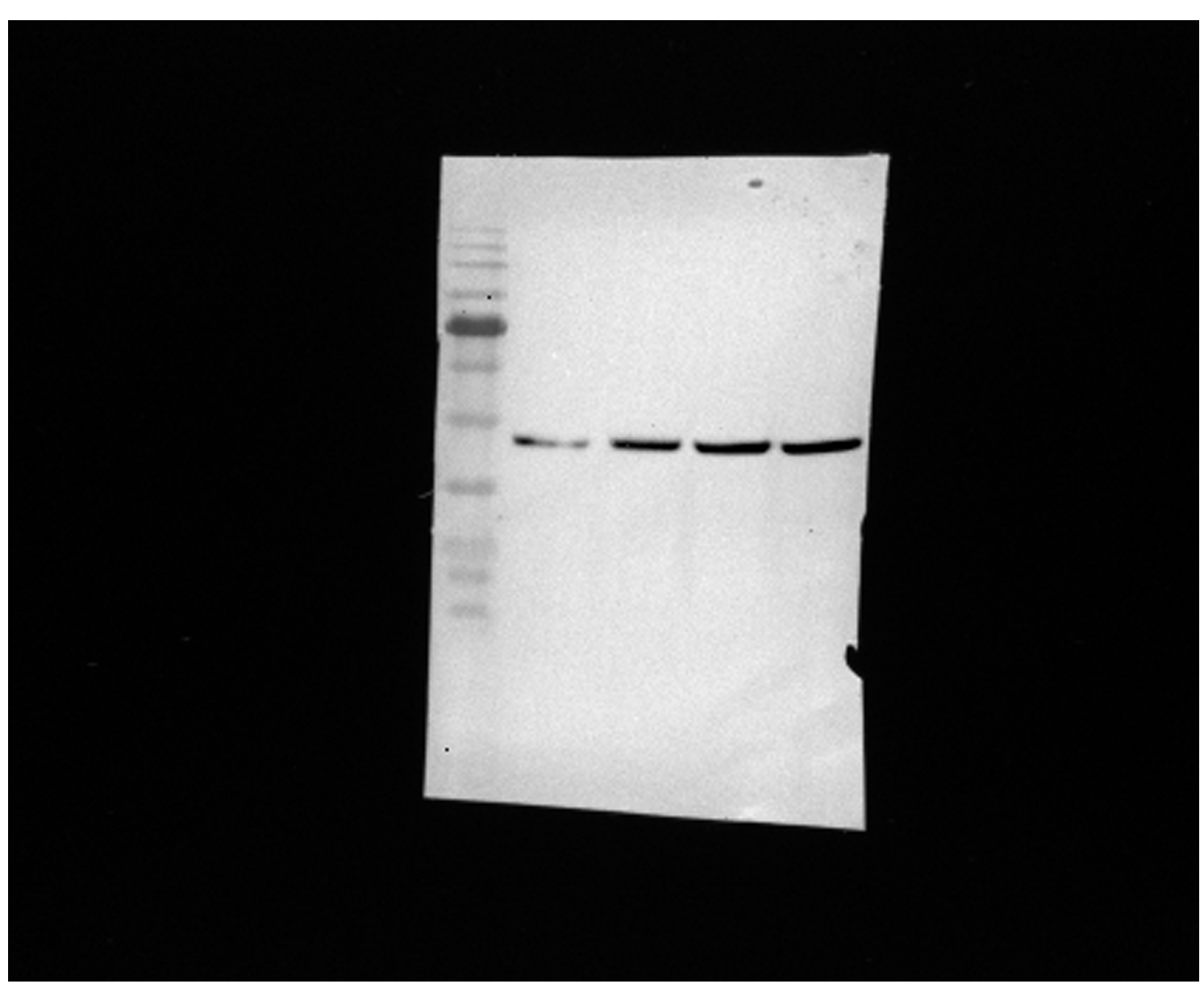

actin

Supplementary Figure 2: Raw images of all the western blots from Figures 1, 4 and 5.

Figure.4 B & C Raw blots

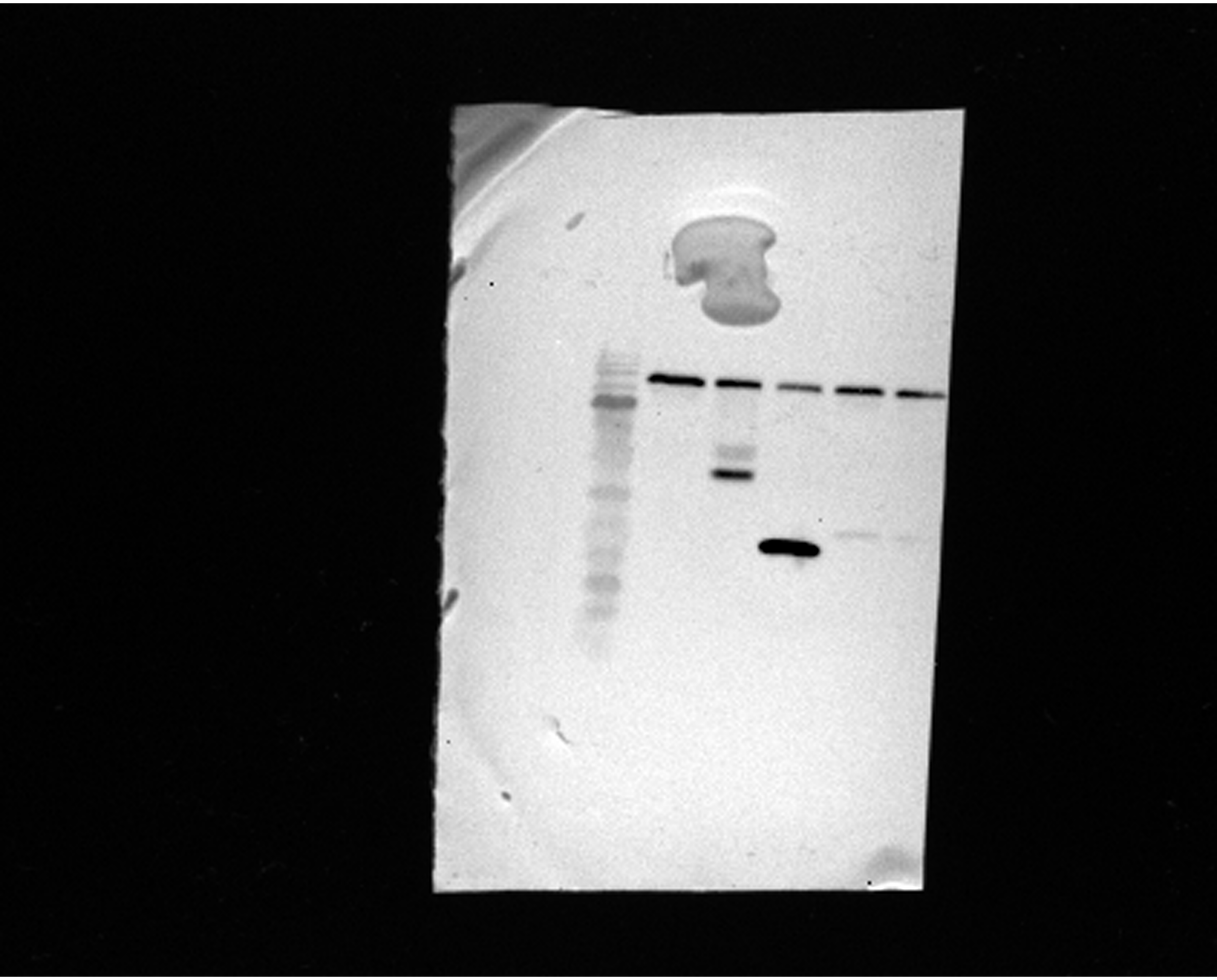

Supplementary Figure 2

Figure 5 Raw Blots

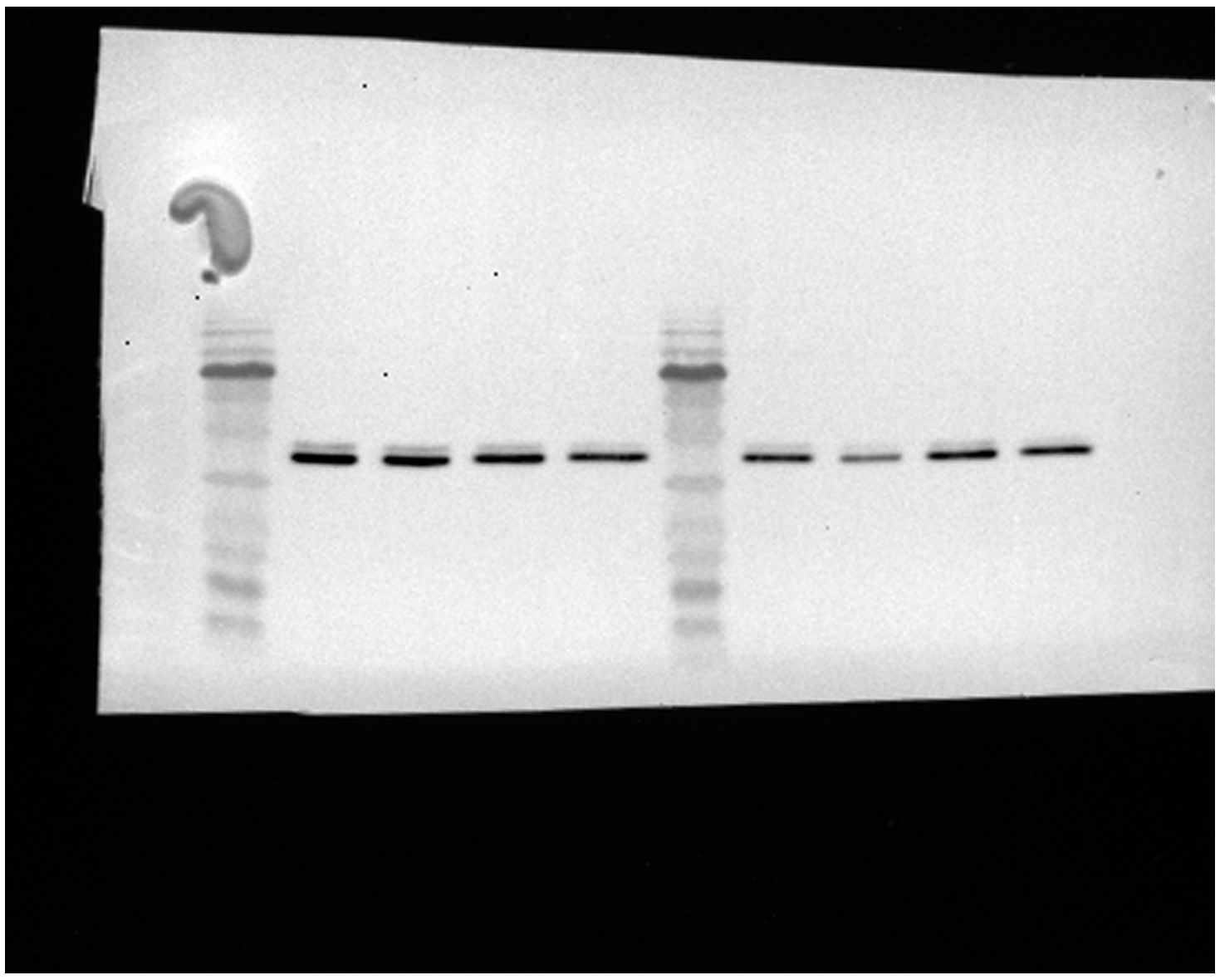

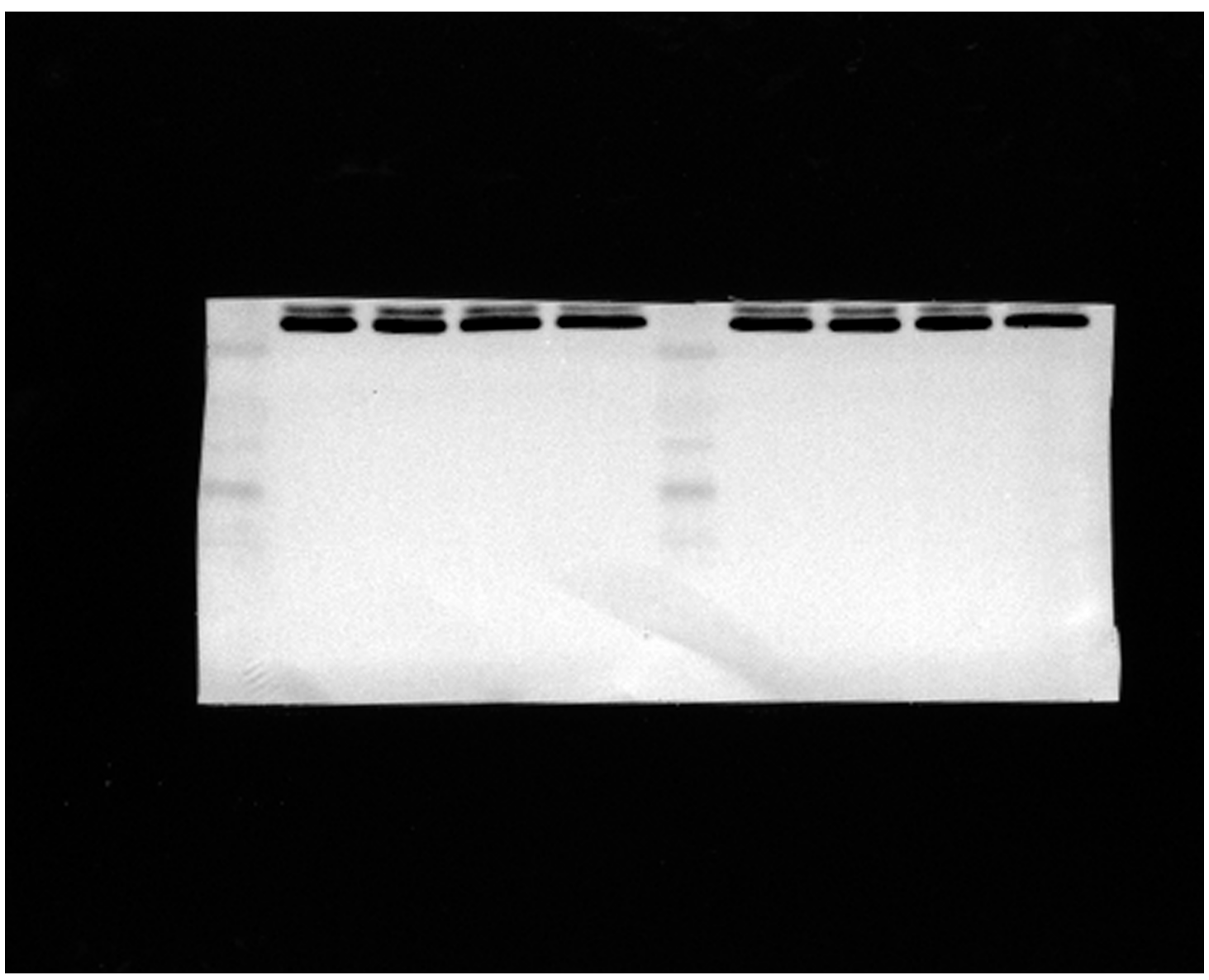

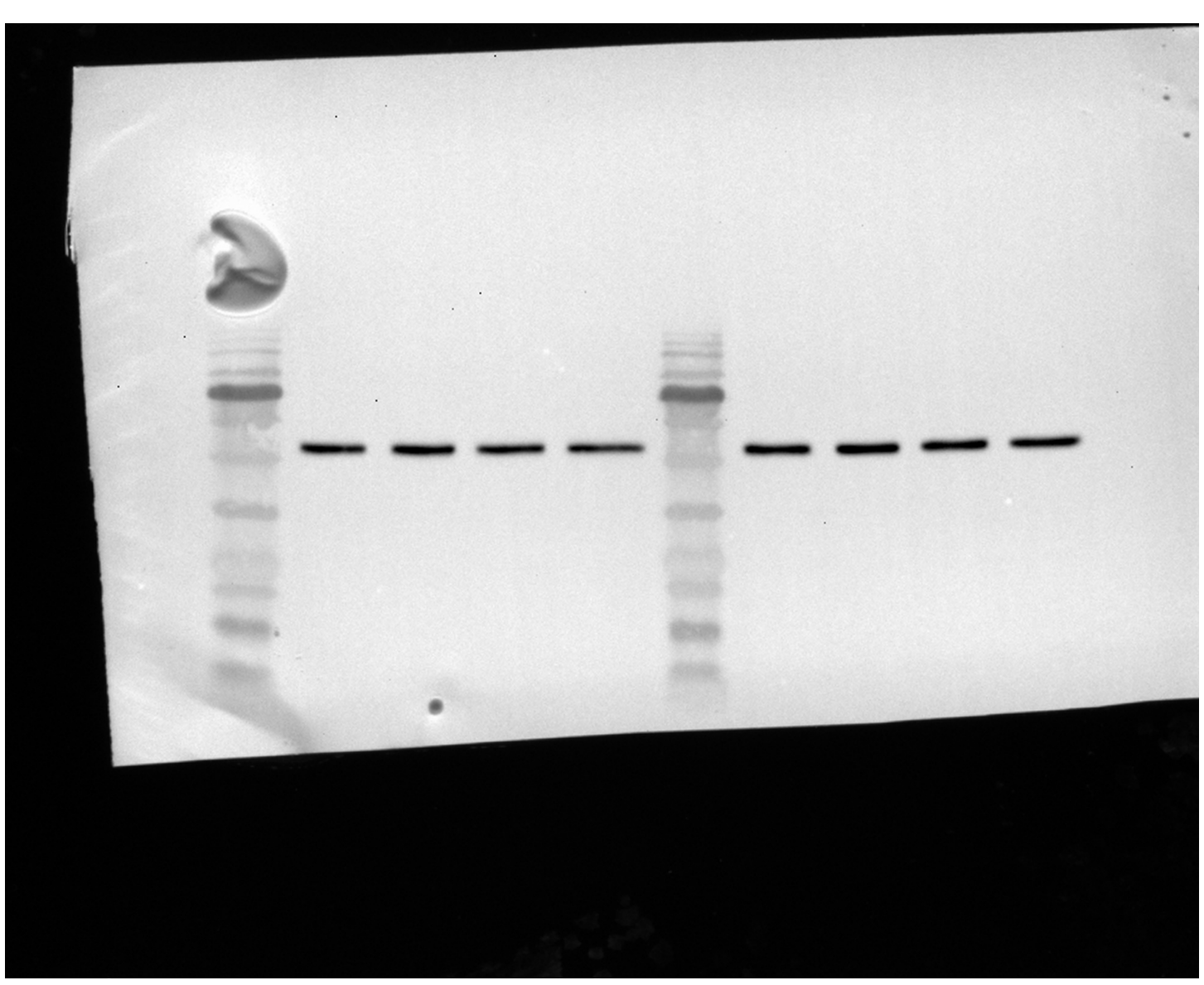

Supplementary Figure 2

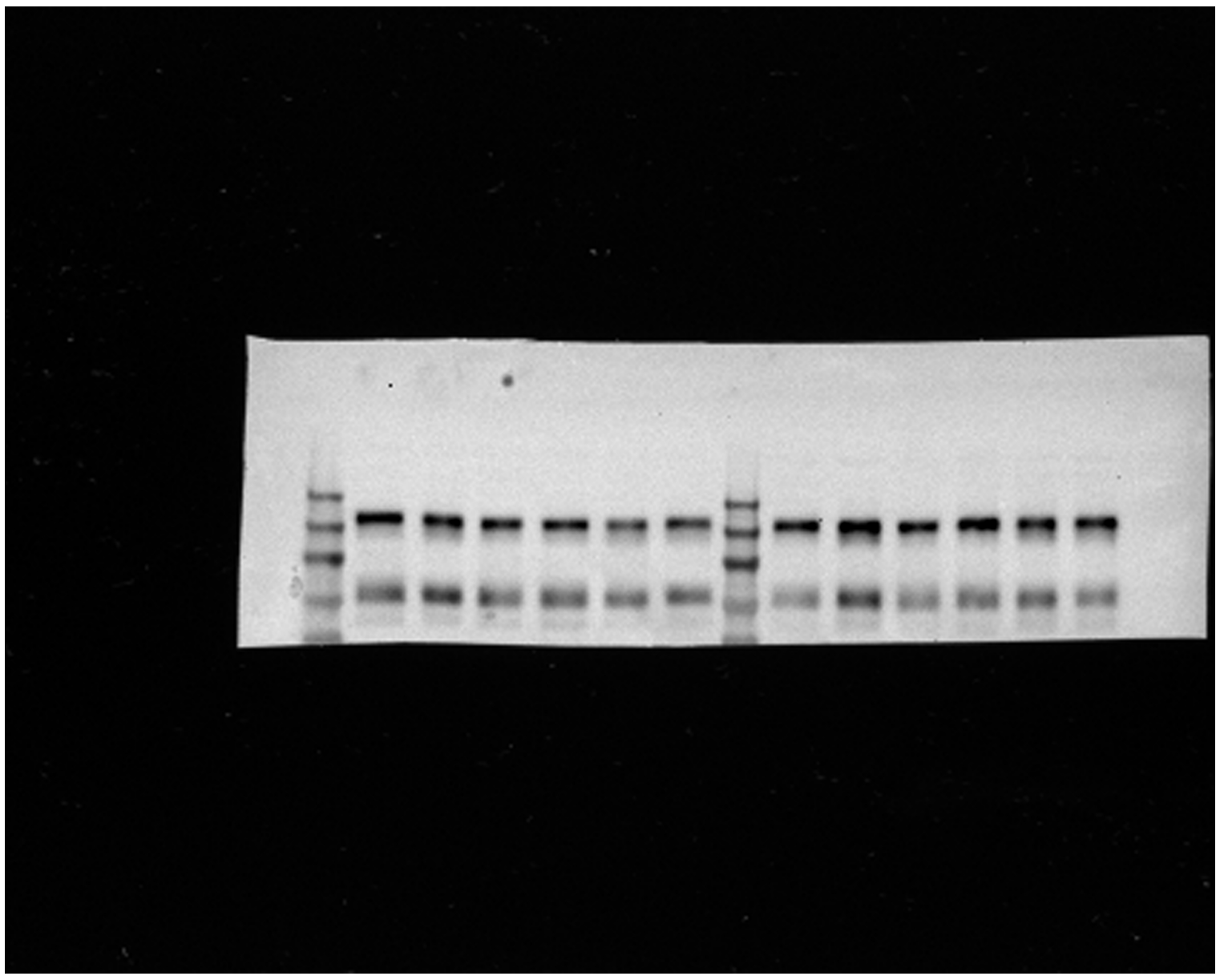

Figure 5 Raw Blots

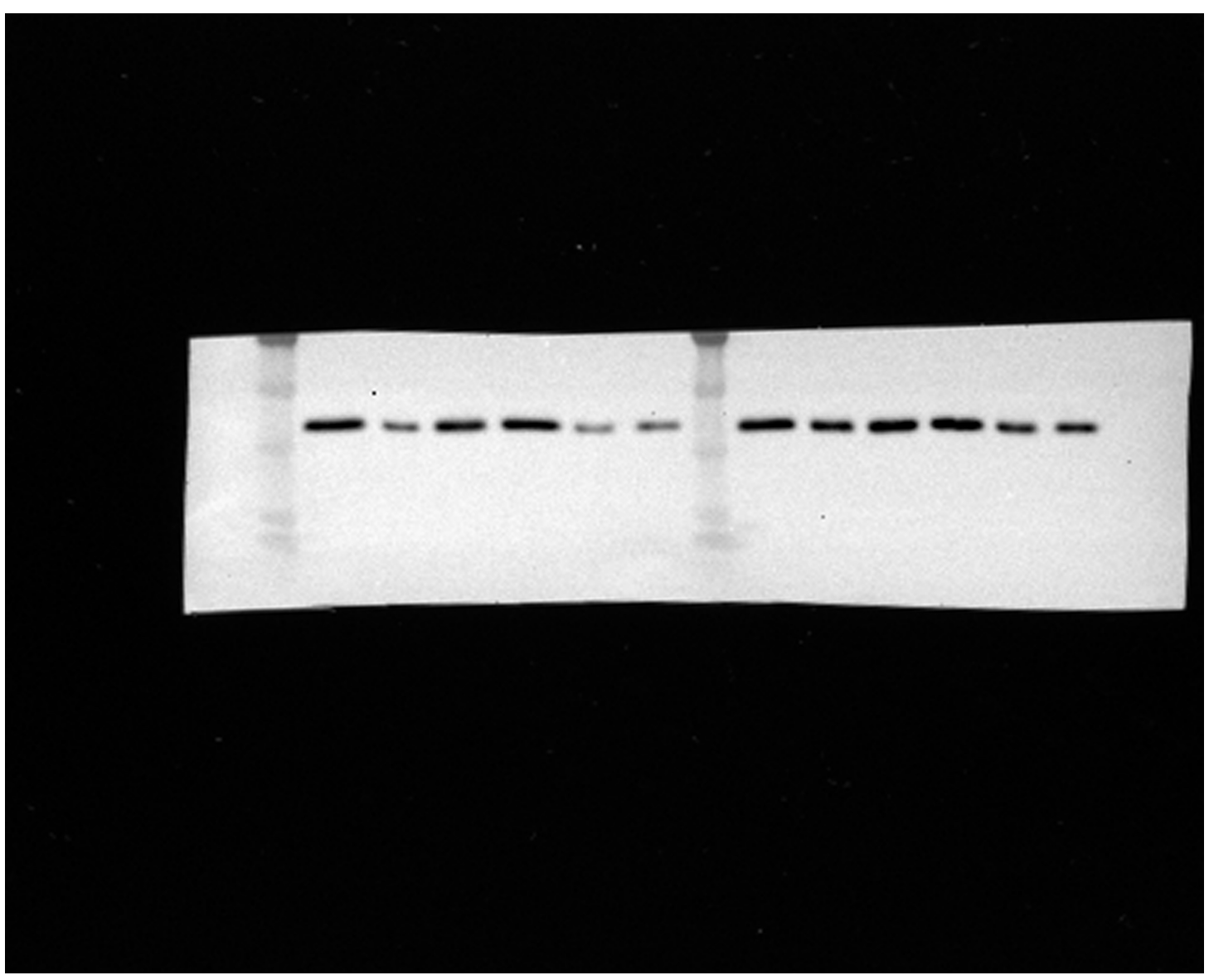

Supplementary Figure 2

Figure 4G Raw Blots

HA-tag for lysates

ctrl SSTR4

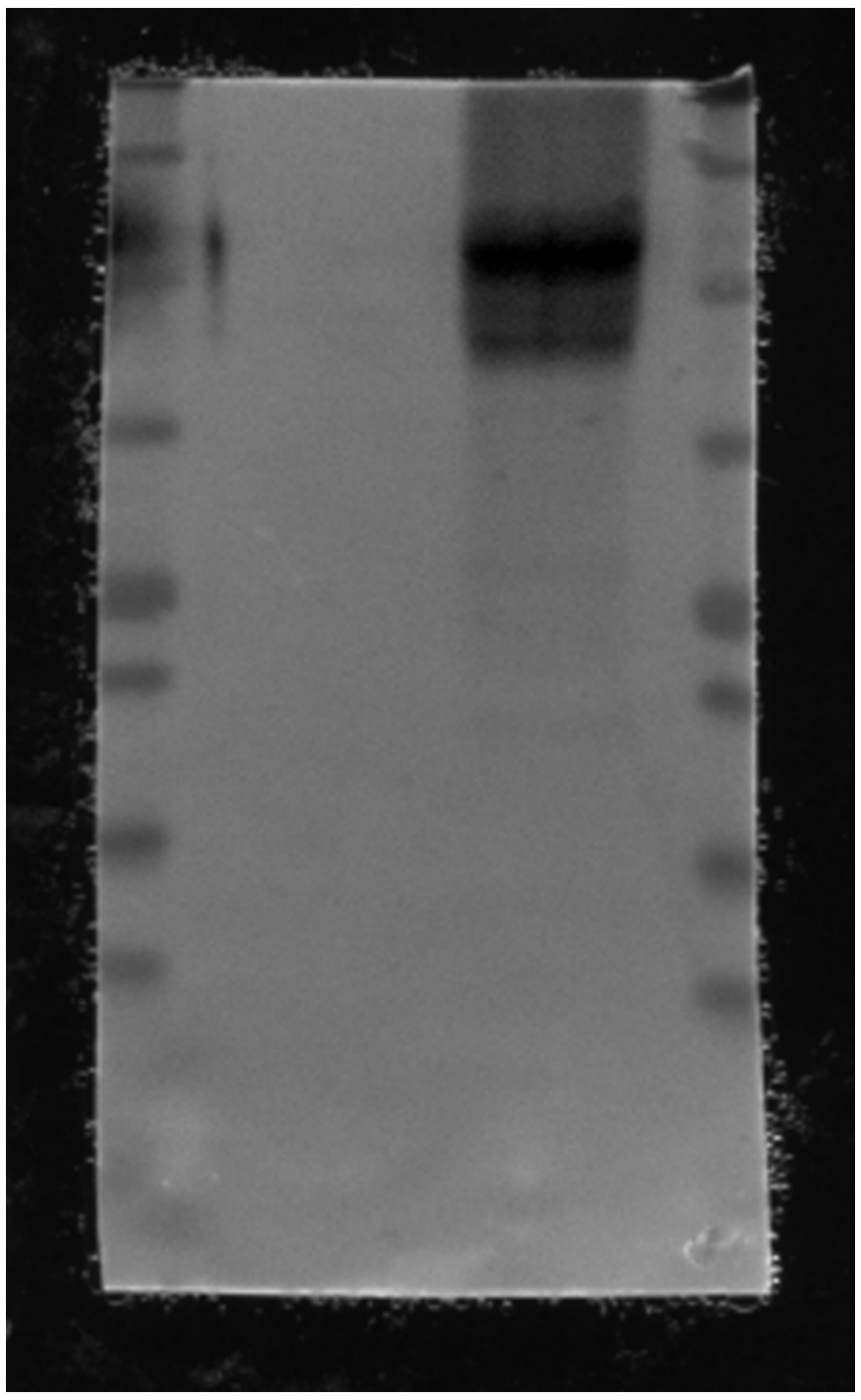

14-3-3 for lysates

ctrl SSTR4

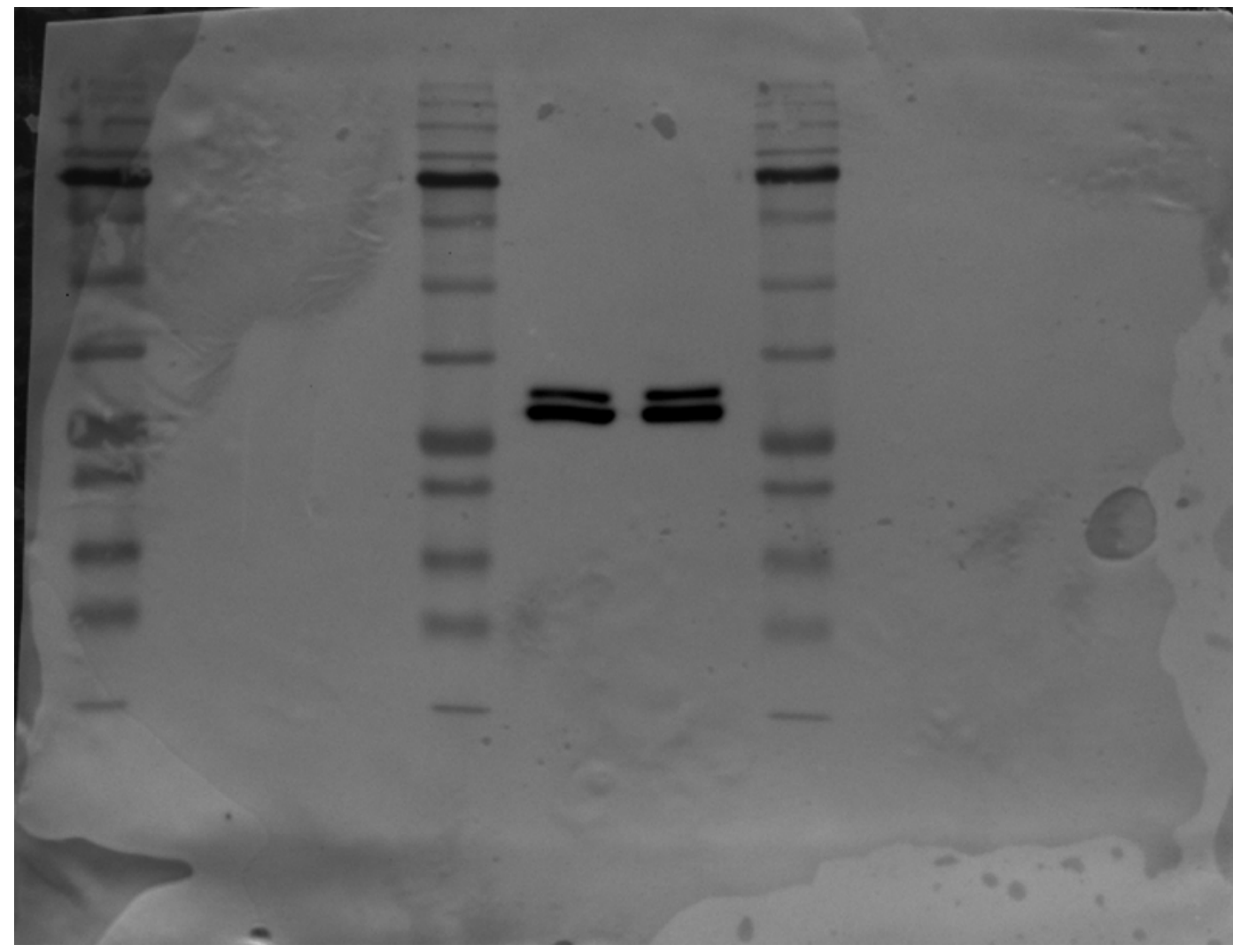

HA-tag for beads 1 sec exposure

ctrl SSTR4

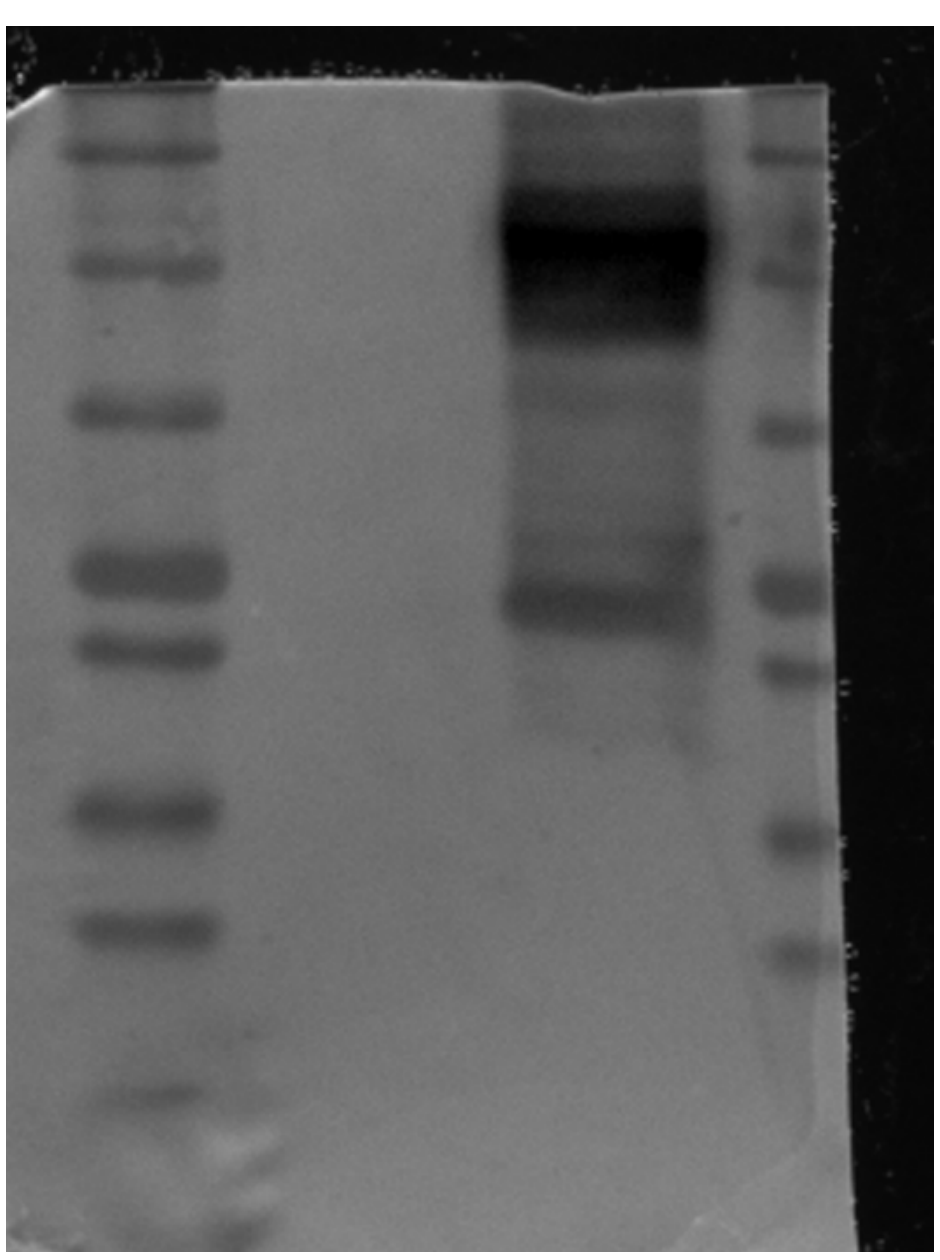

14-3-3 for beads high expo

ctrl SSTR4

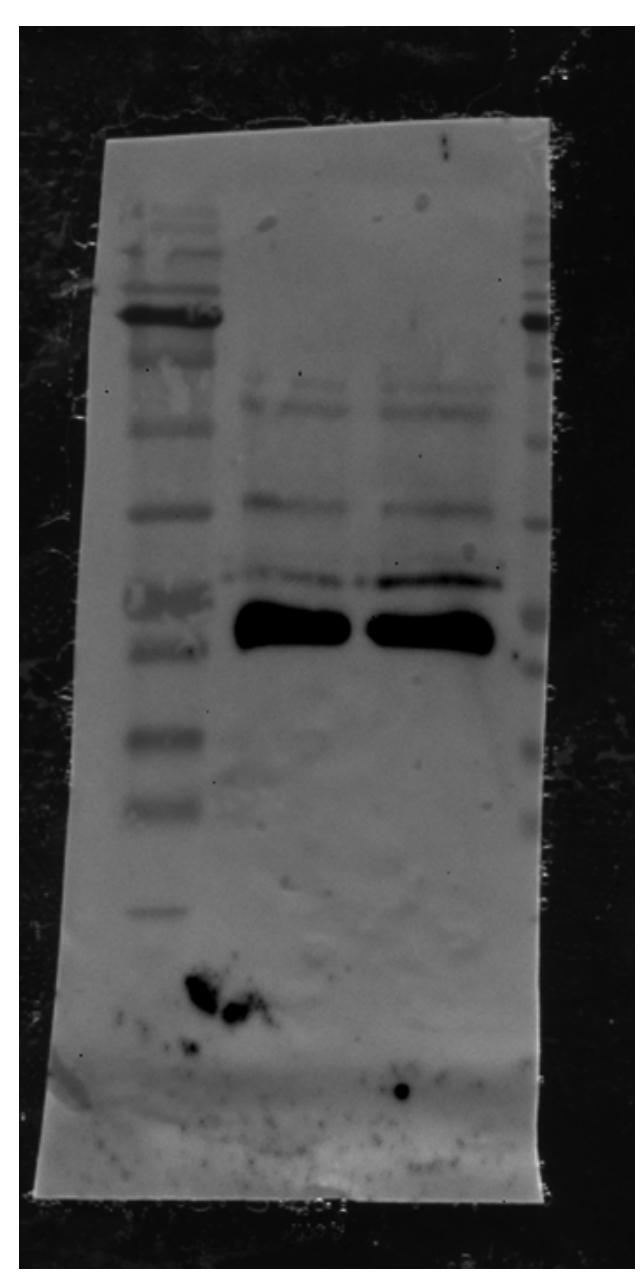

Supplementary Figure 2
